# Robustness of *Helicobacter pylori* Infection Conferred by Context-Variable Redundancy among Cysteine-Rich Paralogs

**DOI:** 10.1371/journal.pone.0059560

**Published:** 2013-03-26

**Authors:** Kalyani Putty, Sarah A. Marcus, Peer R. E. Mittl, Lindsey E. Bogadi, Allison M. Hunter, Swathi Arur, Douglas E. Berg, Palaniappan Sethu, Awdhesh Kalia

**Affiliations:** 1 Department of Biology, University of Louisville, Louisville, Kentucky, United States of America; 2 Department of Biomedical Engineering, University of Louisville, Louisville, Kentucky, United States of America; 3 Department of Biochemistry, University of Zurich, Zurich, Switzerland; 4 Department of Molecular Microbiology, Washington University School of Medicine, St. Louis, Missouri, United States of America; 5 Department of Genetics, the University of Texas MD Anderson Cancer Center, Houston, Texas, United States of America; 6 Molecular Genetic Technology Program, the University of Texas MD Anderson Cancer Center, Houston, Texas, United States of America; Centre National de la Recherche Scientifique, Aix-Marseille Université, France

## Abstract

Deletion of single genes from expanded gene families in bacterial genomes often does not elicit a phenotype thus implying redundancy or functional non-essentiality of paralogous genes. The molecular mechanisms that facilitate evolutionary maintenance of such paralogs despite selective pressures against redundancy remain mostly unexplored. Here, we investigate the evolutionary, genetic, and functional interaction between the *Helicobacter pylori* cysteine-rich paralogs *hcpG* and *hcpC* in the context of *H. pylori* infection of cultured mammalian cells. We find that in natural *H. pylori* populations both *hcpG* and *hcpC* are maintained by positive selection in a dual genetic relationship that switches from *complete redundancy* during early infection, whereby *ΔhcpC* or *ΔhcpG* mutants themselves show no growth defect but a significant growth defect is seen in the *ΔhcpC,ΔhcpG* double mutant, to *quantitative redundancy* during late infection wherein the growth defect of the *ΔhcpC* mutant is exacerbated in the *ΔhcpC,ΔhcpG double* mutant although *the ΔhcpG* mutant itself shows no defect. Moreover, during early infection both *hcpG* and *hcpC* are essential for optimal translocation of the *H. pylori* HspB/GroEL chaperone, but during middle-to-late infection *hcpC* alone is necessary and sufficient for HspB/GroEL translocation thereby revealing the lack of functional compensation among paralogs. We propose that evolution of context-dependent differences in the nature of genetic redundancy, and function, between *hcpG* and *hcpC* may facilitate their maintenance in *H. pylori* genomes, and confer robustness to *H. pylori* growth during infection of cultured mammalian cells.

## Introduction

Gene duplication provides the raw material for functional innovation, and is a source of genetic redundancy and phenotype robustness [1], [2], [3]. Evolutionary theories on the fate of duplicate genes are based on the premise that gene duplication creates functional redundancy thereby relieving selection pressure on one or both gene copies [1], [4], [5]. Thus, mutations normally deleterious to gene function escape purifying selection and, over time, the mutation-containing gene is pseudogenized and lost from the population owing to genetic drift. However, duplicate genes are retained when accumulating mutations cause complementary loss of functional attributes in each copy such that both are required for full functionality [5]. Rarely, some duplicate genes are retained because accumulated mutations confer a new advantageous function [1], [6]. Also, a duplicate gene may be retained because it provides a buffer against deleterious mutations in the ancestral gene [2], [3], [7], [8], [9], [10], [11]. However, because true genetic redundancy is evolutionarily unstable [12], at best transient [8], [13] and only theoretically sustainable on an evolutionary time-scale [3], its contribution to maintenance of duplicate genes remains a subject of intense debate [2], [3], [7], [9], [13].

Sequence-related gene families produced by gene duplications are frequently observed in the genomes of bacteria that maintain long evolutionary associations with their eukaryotic hosts [14], [15]. In such bacteria, gene family expansions are likely associated with host-specific adaptations [14], [15], [16], [17], [18]. Paradoxically, deletion of single genes from expanded gene families often has little or no phenotypic consequence (e.g., on fitness) implying redundancy among paralogs [19], [20]. Conceptually, a lack of a notable phenotype is generally equated with the ability of a system to continue functioning after genetic change, although underlying molecular mechanisms for this mutational robustness remain largely unidentified. In the context of bacterial infection and virulence, whether gene dispensability in expanded gene families reflects the ability of paralogs to functionally compensate each other or a lack of essentiality of ancestral function is not known. Moreover, bacterial genes that are redundant and not under sufficient selection should be rapidly deleted [21], and the evolutionary drive toward small specialist genomes in host-adapted bacteria [16] should exacerbate loss of redundant genes. The mechanisms that facilitate evolutionary maintenance of expanded gene families in bacterial genomes remain largely unexplored.

**Figure 1:**
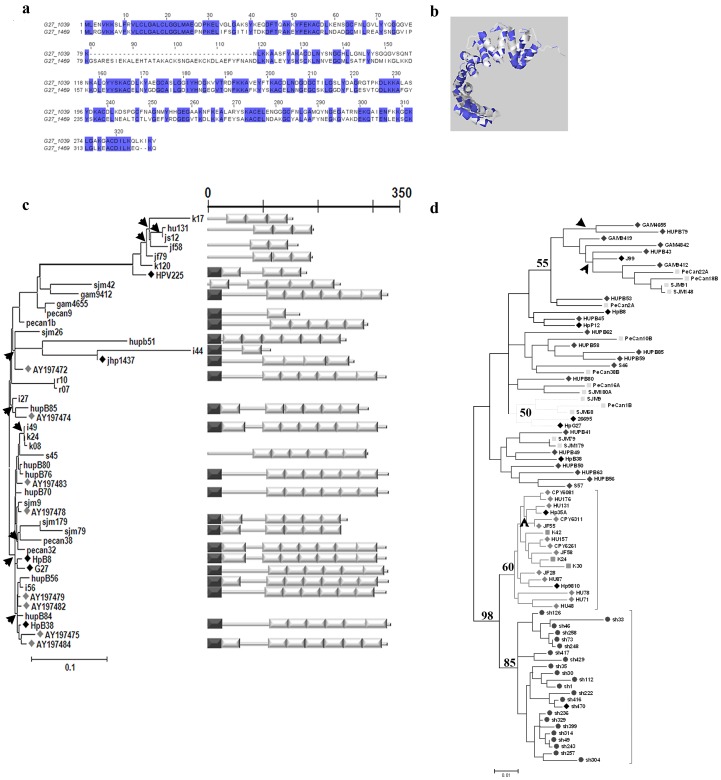
*hcpG* alleles are highly polymorphic whereas *hcpC* alleles are relatively conserved. (**a**) Amino acid alignment of HcpC (G27_1039) and HcpG (G27_1469) from *H. pylori* strain G27MA. Identical amino acids are shaded in blue. (**b**) Mapping of amino acids shared by HcpC and HcpG onto the HcpC crystal structure. The alignment and amino acid mapping was performed using the Jalview multiple sequence editor (version 2.5.1). (**c**) Phylogenetic and corresponding domain architecture analyses of *hcpG* alleles (N = 46). *Left*, The ML phylogeny reconstructed using the TVM+Γ substitution model (**Table S3 in File S1**). Bar = 0.1 nucleotide substitutions per site and the arrowheads indicate *hcpG* lineages that experienced positive selection. Black diamonds, *hcpG* aleles obtained from sequenced *H. pylori* genomes; and grey diamonds, *hcpG* alleles obtained from GenBank. *Right*, domain architectures of representative *hcpG* alleles drawn to scale. The white rectangles are Slrs, and the black rectangles are predicted secretion signal sequences. (**d**) ML phylogeny of *hcpC* alleles (N = 81) reconstructed using the TrN+I+Γ substitution model (**Table S4 in File S1**). The geographic origins of the strains included are listed in **Table S1 in File S1**: black diamonds indicate *hcpC* alleles obtained from sequenced *H. pylori* genomes. Bar = 0.1 nucleotide substitutions per site. Arrowheads indicate *hcpC* lineages that experienced positive selection. Phylogenetic datasets used in generating panels (c) and (d) have been submitted to GenBank® with the following accession numbers: 1) *hcpC* dataset, KC007946–KC008026 and 2) *hcpG* dataset KC008027–KC008064).

*Helicobacter pylori* is an important human pathogen in that it can establish decades-long infections and is the main cause of serious gastric diseases, including ulcers and cancer [22]. Nearly 17% of the *H. pylori* genome is composed of duplicate genes [23], [24], which are categorized into several gene families. Prominent among these is the Sel1-like gene family, which arose from *H. pylori* genome-specific expansion and contains eight diverse, rapidly evolving genes [18]. This gene family is characterized by the presence of modular Sel1-like repeat (*Slr; PFAM entry, PF08238*), which is eukaryotic in origin. Many of the *Slr*-containing genes encode *Helicobacter* cysteine-rich proteins (Hcp), which are highly immunogenic secreted proteins [18], [25], [26], [27], [28], [29], thought to contribute to *H. pylori* infection and pathogenesis. However, little is known about the genetic relationships among *Slr*-containing paralogs or about their functional relevance in the context of *H. pylori* infection or pathogenesis. For example, in *H. pylori* strain HpG27MA HcpG (HpG27_1469) is 53% similar to HcpC (HpG27_1039) (**Fig. 1a** **; Table S6 in File S1)** and predicted to adopt a helical conformation similar to that of HcpC (**Fig. 1b**). However, whereas 1) the *Slr*-gene *hcpG (*also called *hsp12)* is strain-specific, highly polymorphic, and apparently expressed under stress [30], and 2) the divergence of *hcpG* from its closest paralog in the *H. pylori* genome, *hcpC,* is driven by positive selection, indicating functional divergence between the two paralogs [18], *hcpG* appears dispensable to *H. pylori* growth *in vitro* under both normal and stress conditions [30]. Thus, does the dispensability of *hcpG* to *H. pylori* growth reflect that *hcpG* is genetically redundant with *hcpC*? And, does *hcpC* functionally compensate for the lack of *hcpG*? In the present study, we investigated the molecular evolutionary, genetic, and functional relationship between *hcpG* and *hcpC* to explore their relevance to *H. pylori* pathogenesis, and to gain general insight into the mechanisms that maintain duplicate genes in expanded bacterial gene families.

## Results

### *hcpG* is Strain-specific, Highly Polymorphic, and Evolves Rapidly Subject to Positive Selection

This study began with our observation that *hcpG* was present in some fully sequenced *H. pylori* genomes but absent from others (**Table S6 in File S1**). Furthermore, in all genomes analyzed in this study *hcpG* appears to be a single copy gene whose chromosomal context is conserved (**Table S6 in File S1**). To confirm and extend these observations, we first sought to determine whether *hcpG* was present in 166 geographically diverse *H. pylori* isolates (**Fig. S1 and Table S1 in File S1**). Seventy-nine of the isolates were positive for *hcpG* with PCR primers located within genes flanking *hcpG* (**Table S2 in File S1**). PCR primers within *hcpG*-specific conserved internal regions confirmed the absence of *hcpG* among isolates that initially tested negative for *hcpG*. Thus, when present, the chromosomal context of *hcpG* appears conserved. We observed striking variation in the molecular size of *hcpG* in different *H. pylori* isolates (**Fig. S3A in File S1**). Thus, we next examined the molecular basis and effect of size variation on the modular domain architecture of *hcpG* alleles. The complete nucleotide sequences of the 79 PCR products described above revealed that 15 had a non-Slr containing gene in place of *hcpG.* Of the remaining 64 *hcpG* alleles, 28 encoded pseudogenes that contained premature stop codons in their nucleotide sequence introduced mostly via frameshift mutations (**Fig. S3b in File S1**). In *hcpG* nucleotide sequences encoding functional proteins (i.e., >100 amino acids), the number of *Slr* modules ranged from two to seven. Accordingly, the predicted proteins ranged from 107 to 330 amino acids with strikingly different domain architectures (**Fig. 1c**). To better understand *hcpG* evolution, we analyzed 46 unique alleles of *hcpG* phylogenetically. ML analysis revealed no significant geographic clustering of sequences or of different domain architectures (**Fig. 1c**). Next, we estimated selection pressures on individual *hcpG* codons and in branches of the phylogenetic tree using a combination of codon-based models of sequence evolution, ML, and Bayesian methods. Codon models that incorporated positive selection (ω_S_ >>1) within the estimated parameters fit the data significantly better than those that did not (**Table 1**), suggesting that functional *hcpG* alleles are subject to heterogeneous selective pressures. Moreover, Bayesian analysis confidently identified 11 sites under positive selection (Bayesian probability ≥0.99; ω_S_ = 4.46) (**Table 1**). Positive selection was also evident in several branches of the *hcpG* phylogenetic tree (**Fig. 1c** and **Table 1** **; Table S7 in File S1**). Thus, we conclude that *hcpG* is highly polymorphic and present in only 38% of *H. pylori* strains, that some *H. pylori* strains only contain pseudogenized alleles while other strains only contain functional *hcpG* variants, and that positive selection maintains and drives the divergence of extant, functional *hcpG* alleles.

**Table 1:** Maximum-likelihood parameters of selection pressures acting on *H. pylori hcpG* codons.

Model Code	*-InL* *	Tree length	κ^Ŧ^	d_N/_d_S_	Parameter Estimates	D [d.f.]¶	?^2^	P-value	Positively selected sites [Reference sequence: HpI49]
**M0 (1-ratio)**	5242.663	3.2215	4.768	0.325	ω = 0.325	NA	NA	NA	NA
**M1a (Nearly Neutral)**	5130.388	3.4698	4.867	0.341	p0 = 0.742, ω0 = 0.111; p1 = 0.258, ω1 = 1	NA	NA	NA	NA
**M2a (Positive Selection)**	5108.087	3.5508	5.228	0.434	p0 = 0.723, ω0 = 0.117; p1 = 0.256, ω1 = 1; [p_S_] = 0.021, **[ω_S_] = 4.46**	M0 *vs* M2a [3] M1a *vs* M2a [2]	269.152 44.602	<0.0001<0.0001	28S, 30P, 36S, 58K, 66A, 79L, 262A
**M3 (Discrete)**	5105.965	3.551	5.125	0.409	p0 = 0.627, ω0 = 0.083; p1 = 0.334, ω1 = 0.674; [p_S_] = 0.039, **[ω_S_] = 3.365**	M0 *vs* M3 [4] M1a *vs* M3 [4]	273.396 44.846	<0.0001<0.0001	21G. 28S, 30P, 36S, 58K, 66A, 79L, 221L, 224S, 262A, 313K
**M7 (β)**	5135.398	3.4751	4.731	0.321	p = 0.323, q = 0.684	NA	NA	NA	NA
**M8 (β,ω** ***_S_*** **>1)**	5106.781	3.556	5.096	0.405	p = 0.465, q = 1.159; p0 = 0.961, [p_S_] = 0.393, **[ω_S_] = 3.334**	M7 *vs* M8 [2]	57.234	<0.0001	21G. 28S, 30P, 36S, 58K, 66A, 79L, 221L, 224S, 262A, 272S, 313K
**M8a (β, ω_S_ = 1)**	5128.752	3.525	4.659	0.362	p = 1.516, q = 9.983; p0 = 0.733, [p_S_] = 0.267, **ω_S_ = 1**	M8a *vs* M8 [1]	43.942	<0.0001	
**M1bra**	5177.115	3.2376	4.649	NA	Free ω for each lineage	M0 *vs* M1bra	131.1	<0.002	NA

* -*InL*, Log Likelihood Score.

∓, κ, kappa, ratio of transition to transversions.

¶ , D, hierarchical Likelihood Ratio Test statistic and d.f., degrees of freedom.

### *hcpC* Evolution is Characterized by Genomic Conservation and Relatively Stronger Functional Constraint

To better understand the divergence mechanisms of duplicate genes, we next characterized the evolutionary dynamics of *hcpC*. Although there are several *slr* genes encoded in *H. pylori* genome we focus here on *hcpC* because it is the closest paralog of *hcpG* [18]. We found that in contrast with *hcpG*, which was present in only a subset of *H. pylori* strains, *hcpC* was present in all *H. pylori* isolates screened. Complete nucleotide sequence analysis of the *hcpC* alleles from 100 *H. pylori* strains revealed that, unlike *hcpG*, their modular domain architecture was conserved. Also, *hcpC* alleles exhibited less overall nucleotide diversity than did *hcpG* alleles (Table S8 in File S1). Population genetic and ML phylogenetic analyses of 81 unique sequences revealed that unlike *hcpG* alleles, *hcpC* alleles clustered according to their geographic origins (Fig. 1d; Table S9 in File S1). Such geographic clustering is typical of *H. pylori* gene sequences. Because of striking differences in overall genomic conservation and evolution of *hcpG* and *hcpC*, we next examined the selective pressures on individual *hcpC* codons and branches of the *hcpC* phylogenetic tree. We found that *hcpC* codons experienced heterogeneous selective pressures similar to those on *hcpG* codons (Table 2). Corresponding Bayesian analysis confidently (Bayesian probability >0.99) identified 23 *hcpC* codons under positive selection (ω_s_ = 2.73). All 23 sites mapped to the molecular surface of HcpC, some in close proximity to the experimentally identified peptide binding site [31] (Table 2; Fig. S4 in File S1). Thus, unlike *hcpG*, in which large-scale domain architecture changes are positively selected (Fig. 1c) and likely drive gain or loss of protein function [32], we predict that positive selection may simply fine tune *hcpC* functions by modulating its interaction with other bacterial or host proteins [18]. Most notably, although evolutionary rates vary significantly in the *hcpC* phylogenetic tree, few *hcpC* lineages preferentially accumulate non-synonymous substitutions (Fig. 1d, Table S10 in File S1). Moreover, the MacDonald-Kreitman test revealed that *hcpC* evolution was predominantly neutral in different populations (Table S9 in File S1). Note that this test was not useful on *hcpG* sequences because of lack of any fixed differences and geographic partitioning among *hcpG* alleles (Fig. 1C). Thus, we conclude that although divergence of *hcpG* and *hcpC* alleles is driven by positive selection, the intensity of positive selection is stronger on *hcpG (*ω_s_ *hcpG* = 4.46 versus ω_s_ *hcpC* = 2.73). Consequently, functional constraints on *hcpC* are likely stronger and manifest in its overall structural and genomic conservation.

**Table 2:** Maximum-likelihood parameters of selection pressures acting on *H. pylori hcpC* codons.

Model Code	*-InL* *	Tree length	κŦ	d_N/_d_S_	Parameter Estimates	D [d.f.]¶	?^2^		P-value	Positively selected sites [Reference sequence: HpSh30]
**M0 (1-ratio)**	6431.304	3.358	3.448	0.1311	ω = 0.1311	NA	NA		NA	NA
**M1a (Nearly Neutral)**	6137.837	3.447	3.478	0.135	p0 = 0.881, ω0 = 0.018; p1 = 0.119, ω1 = 1	NA		NA	NA	NA
**M2a** **(Positive Selection)**	6127.888	3.499	3.82	0.171	p0 = 0.878, ω0 = 0.018; p1 = 0.102, ω1 = 1; [p_S_] = 0.019, **[ω_S_] = 2.73**	M0 *vs* M2a [3] M1a *vs* M2a [2]		606.832 19.898	<0.0001<0.0001	9L, 43Q, 75Q, 161T, 229M, 248V
**M3 (Discrete)**	6124.796	3.483	3.695	0.155	p0 = 0.794, ω0 = 0.004; p1 = 0.119, ω1 = 0.258; [p_S_] = 0.085, [**ω_S_] = 1.4**	M0 *vs* M3 [4] M1a *vs* M3 [4]		613.016 26.082	<0.0001<0.0001	3E. 8S, 9L, 17A, 32L, 43Q, 46T, 75Q. 86S, 89A, 95D, 161T, 176A, 192G, 210D, 212V. 213A, 229M, 230Q, 248V, 249T, 273L, 281A
**M7 (β)**	6131.577	3.389	3.452	0.117	p = 0.021, q = 0.145	NA		NA	NA	NA
**M8 (β,ω** ***_S_*** **>1)**	6120.872	3.547	3.742	0.162	p = 0.045, q = 0.375; p0 = 0.978 [p_S_] = 0.022, **[ω_S_] = 2.51**	M7 *vs* M8 [2]		21.41	<0.0001	8S, 9L, 17A, 43Q, 46T, 75Q, 86S, 89A, 95D, 161T, 176A, 192G, 210D, 212V, 213A, 229M, 230Q, 248V, 249T, 281A
**M8a (β, ω_S_ = 1)**	6129.478	3.464	3.426	0.127	p = 0.102, q = 3.291; p0 = 0.895, [p_S_] = 0.104, **ω_S_ = 1**	M8a *vs* M8 [1]		17.212	<0.0001	NA
**M1bra**	6324.72	3.362	3.458	NA	Free ω for each lineage	M0 vs M1bra		213.168	<0.004	NA

* -*InL*, Log Likelihood Score.

∓ κ, kappa, ratio of transition to transversions.

¶ D, hierarchical Likelihood Ratio Test statistic and d.f., degrees of freedom.

### *hcpG* is Expressed during *H. pylori* Growth and Infection, but Deletion of *hcpG* does not Cause Defects in *H. pylori* Growth or Fitness

**Figure 2:**
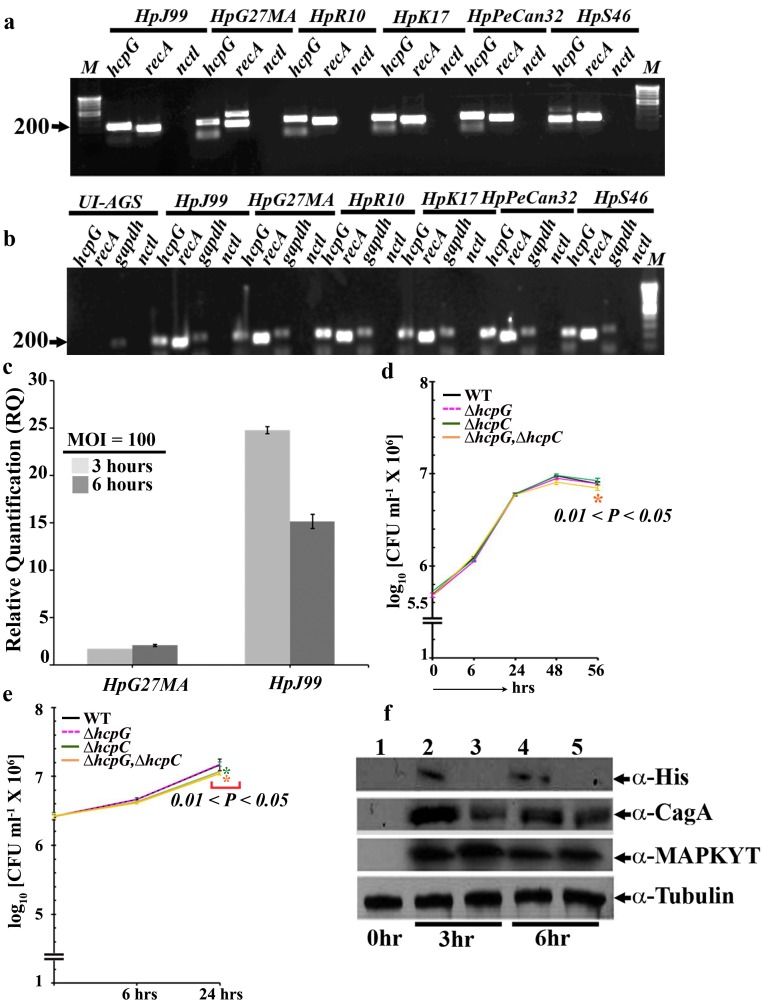
Biological relevance of *hcpG* during *H. pylori* growth and infection. (**a**) RT-PCR amplification of *hcpG* transcripts from 24 h growth in pure broth cultures. *recA*, positive control; nctl, negative control, in which extracted RNA was used as a template for RT-PCR. The geographic origins of the strains used were as follows: HpJ99, United States; HpG27MA, Italy; HpR10, South Africa; HpK17, South Korea; HpPeCan32, Lima, Peru; and HpS46, Spain. (**b**) RT-PCR amplification of *hcpG* transcripts in cultured AGS cells 6 h after infection (multiplicity of infection [MOI] = 100). *gapdh* was included as a positive control for AGS cell transcription in addition to the controls listed in Fig. 3a. UI, uninfected. (**c**). Quantitative RT-PCR analysis of *hcpG* transcripts in cultured AGS cells 3 and 6 h after infection with HpG27MA and HpJ99, respectively. The results show the averages from three experiments (± standard error of the mean). (**d**) Growth curves of WT HpG27MA and *hcpG* and/or *hcpC* derivatives in pure broth cultures; the results show averages from three experiments (± standard deviation [SD]). (**e**). Growth curves of WT HpG27MA strain and *hcpG* and/or *hcpC* deletion derivatives during infection of cultured AGS cells. The results show the averages from five experiments (± SD). (**f**). Verification of HcpG synthesis during infection of cultured AGS cells with the HpG27MA derivative expressing the HcpG::6xHis fusion protein; Lane 1, uninfected AGS cells (control); lanes 2 and 3, 3 h after infection with HpG27MA::HcpG-6XHis and Hp*G27MAΔhcpG*, respectively; lanes 4 and 5∶6 h after infection with HpG27MA::HcpG-6XHis and Hp*G27MAΔhcpG*, respectively. α-Tubulin was used as a measure for equal gel loading. CFU, colony-forming unit.

To test the functional essentiality of *hcpG*, we determined whether it was expressed in broth cultures during *H. pylori* growth and cultured AGS cells during *H. pylori* infection. Under both conditions, we detected robust *hcpG* mRNA transcripts from diverse strains (**Fig. 2a and 2b**). We next quantified the *hcpG* mRNA transcripts during infection of cultured AGS cells with the *H. pylori* strains HpG27MA and JHp99, both of which have been extensively characterized. This analysis revealed that 3 and 6 h after infection with HpG27MA, *hcpG* expression was up-regulated 2.0 and 2.5 fold, respectively. However, following infection with JHp99, *hcpG* expression was up-regulated 25-fold at 3 h but then down-regulated to 16-fold at 6 h (**Fig. 2c**). These data suggested that *hcpG* is expressed *in vitro* and during infection and that its expression is likely to be differentially regulated in distinct *H. pylori* strains.

To assess the contribution of *hcpG* to *H. pylori* growth and infection, the growth of the *ΔhcpG* mutant strain in broth culture, and then during infection of cultured AGS cells was compared with that of the WTHpG27MA strain. This comparison revealed that the *ΔhcpG* mutant had no significant growth defect in either broth culture or during infection of cultured AGS cells (**Fig. 2d and 2e**).

We next determined whether the mild up-regulation of *hcpG* transcript expression seen in HpG27MA resulted in protein production. For this purpose we reverse engineered the *ΔhcpG* mutant strain by replacing the *rpsL,catR* cassette with a *hcpG*::6xHis fusion assembly (**Fig. S2 in File S1**). We detected the HcpG::6xHis fusion protein following infection of AGS cells with the HpG27MA::*hcp-6xhis* strain (**Fig. 2f**). Furthermore, the *ΔhcpG* mutant and HpG27MA::*hcp-6xhis* strains both triggered bacterial CagA translocation and subsequent activation of cellular MAPK (ERK2) in AGS cells suggesting that both strains initiated normal infection-induced signaling events [33] (**Fig. 2f**).

**Figure 3:**
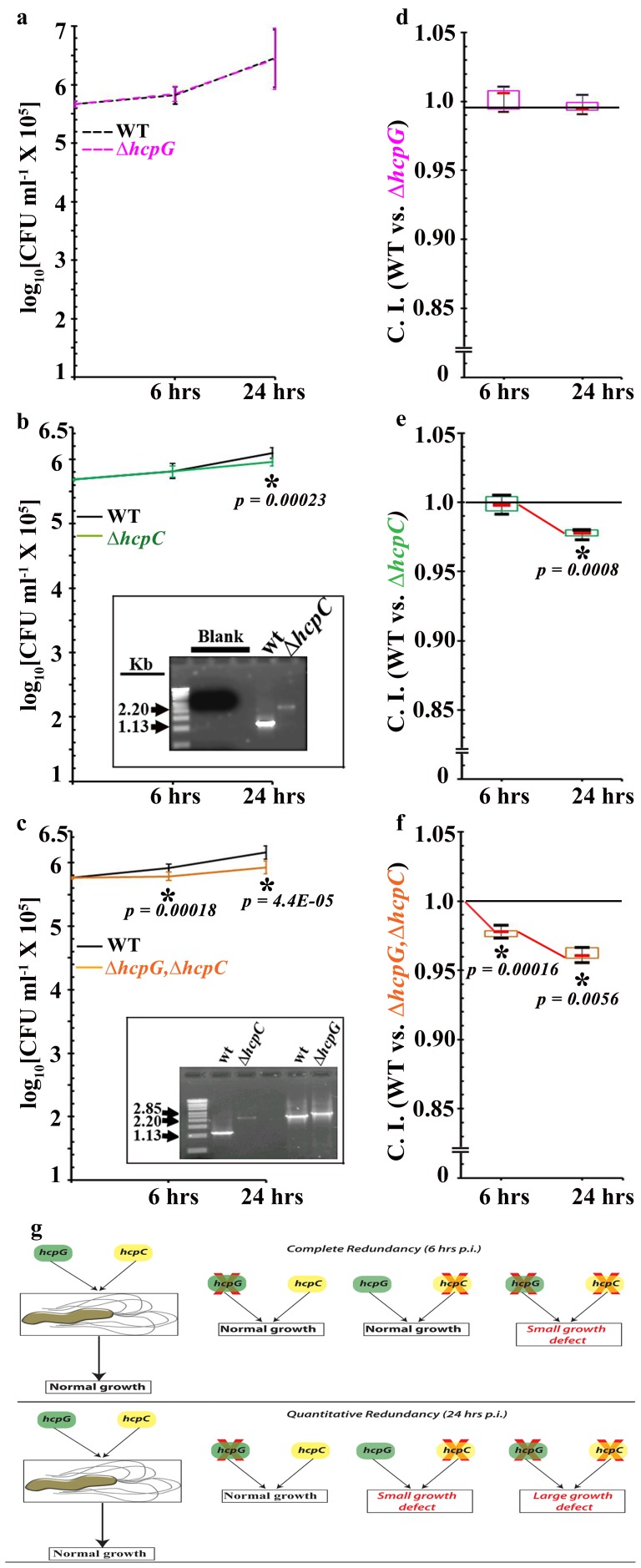
*hcpC* and *hcpG* can be completely or quantitatively redundant. (**a**) Growth curves for the *ΔhcpG* mutant derivative and HpG27MA WT strain. (**b**) Growth curves for the *ΔhcpC* mutant and HpG27MA WT strains. *Inset:* PCR performed to verify the presence of both strains in the infection mix. (**c**) Growth curves for the *ΔhcpG*,*ΔhcpC* double mutant and HpG27MA WT strains. *Inset,* PCR performed to verify the presence of both strains in the infection mix. (**d**) Unbiased box and whisker plot representation of the CI for the *ΔhcpG* mutant. (**e**) CI measurements for the *ΔhcpC* mutant. (**f**) CI measurements for the *ΔhcpC,ΔhcpG* double mutant. The median reduction in the CI from 6 to 24 h after infection is indicated by the red connector. The asterisks indicate significant *P*-values. The results show the averages from five experiments (± SD). (**g**) Interpretation and representation of complete and quantitative redundancy of *hcpG* and *hcpC* in competition assays.

We next assayed the growth and fitness of the *ΔhcpG* mutant strain relative to that of the WT strain during competition in broth culture or during infection in cultured AGS cells. These competition assays revealed no significant reduction in growth or fitness of the *ΔhcpG* mutant strain in broth culture or during infection of cultured AGS cells (**Figs. 3a and 3d**; **Figs. S5a and S5d in File S1**). Thus, data from absolute and relative measures of fitness suggest that *hcpG* is dispensable to *H. pylori* growth and infection even though *hcpG* is expressed *in vitro* and during infection in cultured AGS cells.

### *hcpG* and *hcpC* are Completely and Quantitatively Redundant Specifically during Infection

We next asked whether the dispensability of *hcpG* indicated lack of essentiality of its ancestral function or some form of genetic buffering by its paralog, *hcpC*. To answer this question, we engineered two mutant *H. pylori* strains for use in growth and fitness assays: 1) HpG27MA*ΔhcpC*, and 2) the HpG27MA*ΔhcpG,ΔhcpC* double mutant [See supplementary methods in **File S1**]. We found that compared to the WT HpG27MA strain, the *ΔhcpC* mutant had no growth defects in pure broth culture (**Fig. 2d**). However, we observed a small but significant growth defect in the *ΔhcpC* mutant in cultured AGS cells 24 h after infection (0.01<P<0.05; **Fig. 2e**). The *ΔhcpG,ΔhcpC* double mutant exhibited no defects for up to 48 h of growth in pure broth culture, but measurements at 56 h revealed a small but significant growth defect (0.01<P<0.05; **Fig. 2d**). Similar to the *ΔhcpC* mutant, the *ΔhcpG,ΔhcpC* double mutant had a small growth defect in cultured AGS cells 24 h after infection (0.01<P<0.05; **Fig. 2e**). These data suggest that *hcpC* may be required for optimal *H. pylori* growth late during infection of cultured AGS cells and that deletion of both *hcpG* and *hcpC* is mildly deleterious for growth in late broth culture and during late infection. Thus, collectively these results indicate a possible genetic interaction between *hcpG* and *hcpC*.

To confirm and clarify the nature of the genetic interaction between *hcpG* and *hcpC*, we co-cultured the *ΔhcpC* and *ΔhcpG,ΔhcpC* mutants, respectively, with the WT strain in broth cultures and during infection in cultured AGS cells. The *ΔhcpC* mutant exhibited no growth defects in competition assays in broth culture **(Fig. S5b and S5e in File S1)** or in cultured AGS cells 6 h after infection. However, unlike the *ΔhcpG* mutant, the *ΔhcpC* mutant had a significant growth defect and reduced fitness relative to that of the WT strain in cultured AGS cells 24 h after infection (**Fig. 3b and 3e**). Strikingly, the *ΔhcpG,ΔhcpC* double mutant, unlike the *ΔhcpC* and *ΔhcpG* single mutants, had significant fitness reduction relative to that of the WT strain in cultured AGS cells 6 h after infection (**Fig. 3c and 3f**). Moreover, the *ΔhcpG,ΔhcpC* double mutant showed significant fitness reduction, even more than that observed for the *ΔhcpC* mutant, in cultured AGS cells 24 h after infection (**Fig. 3c and 3f**). Similar to the *ΔhcpG* and *ΔhcpC* mutants, the *ΔhcpG,ΔhcpC* double mutant experienced no fitness reduction when co-cultured with the WT strain in broth culture (**Fig. S5c and S5f in File S1**). Thus, we identify two categories of genetic interactions between *hcpG* and *hcpC* depending on the temporal context of *H. pylori* infection (**Fig. 3g**). First, during early infection, *hcpG* and *hcpC* are *completely redundant* in that disrupting either gene alone has no effect on *H. pylori* growth or fitness, but disrupting both genes causes significant reduction in *H. pylori* growth fitness. Second, during late infection *hcpG* and *hcpC* are *quantitatively redundant* in that the fitness phenotype of the *ΔhcpC* mutant is exacerbated in the *ΔhcpG,ΔhcpC* double mutant. Thus, *hcpG* and *hcpC* are coupled in a redundant relationship that differs depending on the temporal context of the infection. We conclude that context-dependent redundant relationships between *hcpG* and *hcpC* contribute significantly to the mutational robustness of *H. pylori* growth during infection and likely contribute to the retention of *hcpG* along with *hcpC* in select *H. pylori* genomes.

### Molecular Role of *hcpG* and *hcpC:* Infection-induced Signaling-dependent Regulation of *H. pylori* HspB/Hsp60/GroEL Export

**Figure 4:**
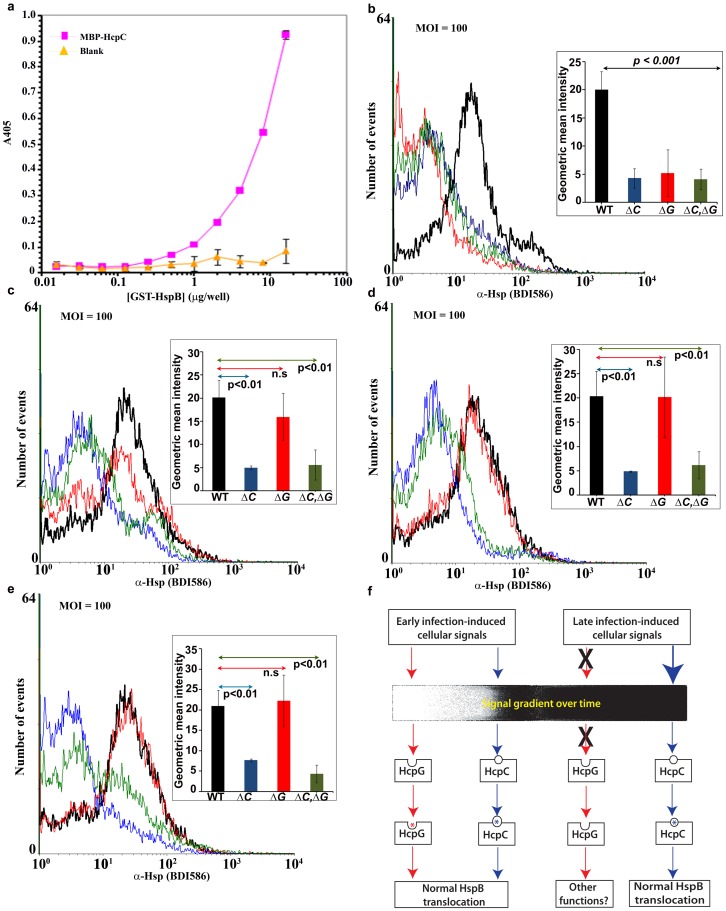
Modulation of HspB translocation by HcpG and HcpC during *H. pylori* infection. (**a**) ELISA performed to determine the specificity of HcpC-HspB interaction, *in vitro*. (**b–e**) FACS analyses of HspB translocation in single- and double *hcp* gene mutants and in the parent HpG27MA WT strain (b) 3, (c) 6, (d) 12, and (e) 24 h after infection of serum-starved confluent AGS cell cultures. Overlapping histograms of mean geometric intensity from individual infections were generated using the WINMDI software program (version 2.9; Joseph Trotter, Scripps Research Institute, LA Jolla, CA). x-axis, α-HspB fluorescence; y-axis, number of events measured for each sample (10^4^ cells/sample). A shift of fluorescence to the left indicates reduced intensity. The FACS parameters are listed in supplementary methods available in File S1. *Inset*, bar graphs showing the geometric mean intensity of α-HspB fluorescence at each time point after infection with mutant and WT strains, respectively. The strains are color-coded as follows: Black, WT; blue, *ΔhcpC*; red, *ΔhcpG*; and green, *ΔhcpG,ΔhcpC*. The results show the averages from three experiments (± SD). (**f**). Model of observed functional interactions of HcpC, HcpG, and HspB translocation depending on the temporal context of the infection.

We next considered molecular mechanisms underlying the genetic redundancy of *hcpG* and *hcpC.* Using a combination of co-immunoprecipitation and mass-spectrometry the *Helicobacter* HspB/Hsp60/GroEL chaperone was identified as a potential interacting partner of HcpC [34]. Using ELISA we confirmed that HcpC can bind directly to HspB (**Fig. 4a**). HspB/Hsp60/GroEL is an essential chaperone that is cytoplasmic in most bacteria except *H. pylori* in which it can also be translocated to the bacterial surface or extracellular milieu [35], [36]. The translocated HspB protein then associates with the UreB subunit of the *H. pylori* urease complex and contributes to *H. pylori* pathogenesis via multiple pathways [37], [38]. The mechanism of HspB translocation is controversial, although it is likely translocated actively [36]. Because *Slr-*containing proteins facilitate protein-protein interactions, we hypothesized that the redundant partners HcpC and HcpG may mediate or modulate HspB translocation.

To determine whether HspB translocation is affected by *hcpG* or *hcpC,* we analyzed HspB expression in *unpermeabilized* WT and mutant *H. pylori* strains in broth cultures and during infection of AGS cells using fluorescence-activated cell sorter (FACS) analysis. We found that 3 h after infection, HspB fluorescence was significantly reduced in the *ΔhcpG* and *ΔhcpC* single mutants and in the *ΔhcpG,ΔhcpC* double mutant (**Fig. 4b**). Strikingly, 6 h after infection with the, HspB fluorescence in the *ΔhcpG* mutant strain recovered nearly to the same level as seen with WT strain (**Fig. 4c**); by 12 and 24 h after infection, the *ΔhcpG* mutant and WT strain demonstrated similar HspB fluorescence levels (**Fig. 4d and 4e**). In contrast, HspB fluorescence remained significantly reduced throughout the infection in the *ΔhcpC* mutant and *ΔhcpG,ΔhcpC* double mutant (**Figure 4b–4e**). Importantly, parallel experiments with pure broth cultures maintained for up to 56 h revealed similar HspB fluorescence levels in the WT and mutant strains (**Fig. S6 a–c in File S1**). Thus, these data demonstrate apparent modulation of HspB translocation specifically in response to infection-induced signals.

**Figure 5:**
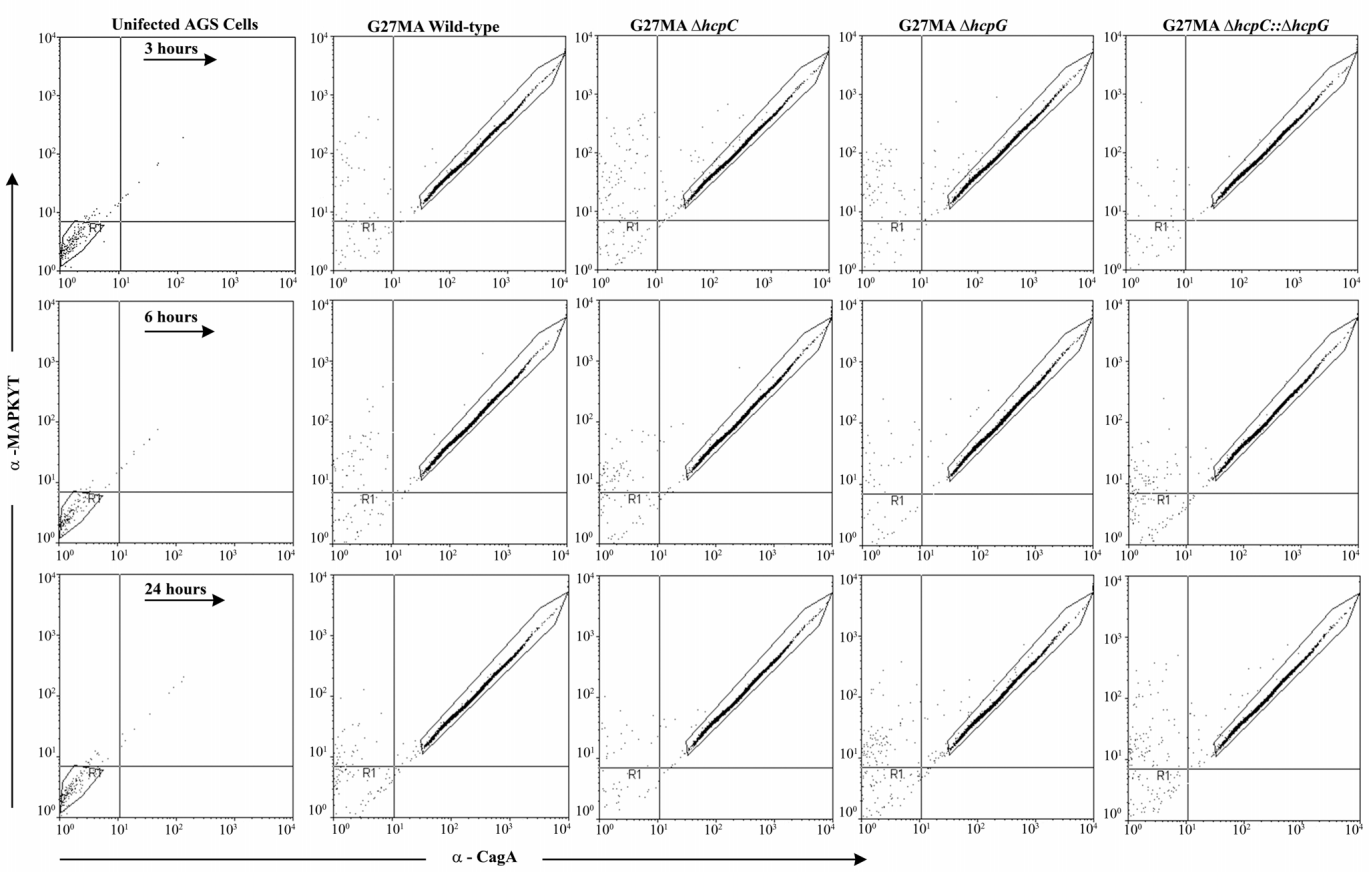
Deletion of *hcpG* and/or *hcpC* does not alter independent infection-induced signaling events. CagA and activated MAPK (ERK2) expression in *hcp* mutants, WT G27MA and uninfected AGS cells over the course of infection. A shift in the fluorescence to the upper right quadrant in AGS cells infected with either WT or *hcpC* or *hcpG* mutants compared with uninfected AGS cells indicates increased CagA and activated-MAPK expression after infection. Results are representative of three experiments.

To confirm that the modulation of HspB expression did not reflect generalized disruption of infection-induced signaling events in response to deletion of *hcpG* and/or *hcpC*, we monitored CagA and MAPK (ERK2) expression levels in infected *permeabilized* AGS cells using FACS analysis. We found that the mutant and WT strains described above all similarly triggered the release of bacterial CagA accompanied by activation of cellular MAPK (**Fig. 5**). Thus, *hcpG* and *hcpC* specifically modulate HspB expression whereas independent *H. pylori* infection-induced signaling events remain unaffected.

**Figure 6:**
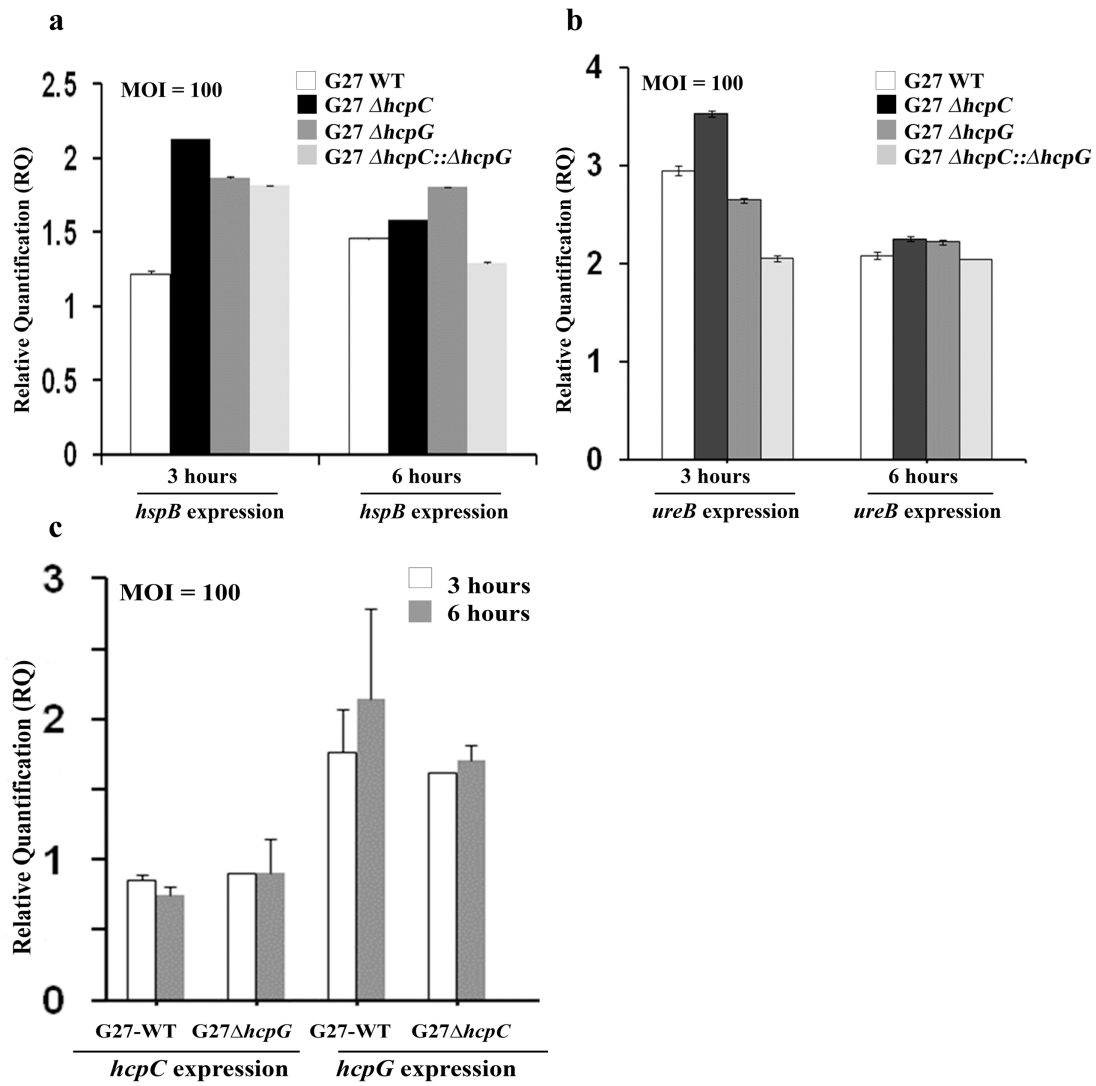
*hcpC* and *hcpG* are transcriptionally uncoupled, and do not affect *hspB* or *ureB* expression during *H. pylori* infection of AGS cells. (a) Fold change in *hspB* expression levels 3 and 6 h after infection with WT and *hcp* mutant bacteria. (b) Fold change in *ureB* expression levels 3 h and 6 h after infection with WT and *hcp* mutant bacteria. A P>0.1 (not significant) was observed using a one-way ANOVA test in comparisons of observed differences. (c) Fold change in *hcpC* expression levels in the *ΔhcpG* mutant of G27MA and in *hcpG* expression levels in the *ΔhcpC* mutant 3 and 6 h after infection. A P>0.1 (not significant) was observed using a one-way ANOVA test in comparisons of observed differences.

We next asked whether *hcpG* and *hcpC* affected the transcriptional regulation of *hspB*, which in turn may alter HspB expression. Real-time (RT) PCR analysis revealed no significant alterations in *hspB* expression levels in the mutant and WT strains following infection of AGS cells (**Fig. 6a**). Because HspB is known to associate with the UreB subunit of the urease complex we also measured the *ureB* expression levels to rule out indirect causes of altered HspB expression. We found no significant alteration in *ureB* expression in the mutant and WT strains at equivalent intervals during infection (**Fig. 6b**). Thus, these data suggest that altered HspB fluorescence does not result from modulation of *hspB* expression in *hcpG* or *hcpC* mutants.

We next determined whether the deletion of *hcpG* resulted in the upregulation of *hcpC* expression, which, if true, could explain the apparent normalization of GroEL/HspB fluorescence to WT levels in the *ΔhcpG* mutant (**Fig. 4c–4e**). We found that *hcpC* expression in the *ΔhcpG* mutant was not significantly affected (**Fig. 6c**). Similarly, deletion of *hcpC* had no significant effect on *hcpG* expression. Together these data suggest that *hcpC* and *hcpG* are transcriptionally uncoupled.

Taken together, our results reveal two categories of functional interactions between *hcpG* and *hcpC* depending on the temporal context of *H. pylori* infection (**Fig. 4f**). First, during early infection, *hcpG* and *hcpC* are both essential for optimal HspB translocation and that neither of them can functionally compensate for deletion of the other gene. Thus, *hcpG* and *hcpC* are selected independently to perform HspB translocation. Second, during middle to late infection, *hcpC* alone is necessary and sufficient for optimal HspB translocation whereas *hcpG* is not required for it. Given the quantitatively redundant fitness phenotypes exhibited by *hcpG* and *hcpC,* our results suggest that these two genes are likely important because of their capacity to perform distinct functions. We conclude that *hcpG* and *hcpC* partially overlap in their function but lack the generic functional backup capacity expected among genetically redundant paralogs.

## Discussion

### Simultaneous Occurrence of Different Types of Genetic Interactions between *hcpG* and *hcpC* in *H. pylori* Infection

Genetic buffering interactions are most commonly studied by measuring the fitness of an organism under standard laboratory growth conditions, in which the spatial and temporal flux in the organism’s interactions with its environment is inherently underestimated. Moreover, in most cases, the molecular functions underlying such genetic interactions remain relatively unexplored. In our study, by taking into account the temporal context of *H. pylori* infection modeled in cultured mammalian cells, we uncovered the simultaneous occurrence of different types of genetic buffering interactions between the *H. pylori* paralogs *hcpG* and *hcpC*. A recent study reported that multiple genetic interactions in yeast paralogs conferred robustness to yeast signaling and regulatory networks [11]. In bacteria, studies of duplicate genes have historically focused on tandem gene duplications (gene amplification) but rarely on expanded gene families [39]. To the best of our knowledge, no previous reports have generally described multiple genetic buffering interactions among duplicate genes from expanded bacterial gene families in the context of pathogen-host interaction. Moreover, our data caution against the prevalent notion that absence of phenotypes upon deletion of single genes from expanded gene families reflects either compensation of function by other paralogs or lack of essential function of paralogs in mediating pathogen-host interactions.

### Selective Advantage of *hcpG* and *hcpC* in *H. pylori* Infection

Previous studies have proposed that maintenance of redundant paralogs can have several selective advantages, including mutational robustness [2], [3], [7], robustness against random fluctuations in gene expression [8], [9] and robustness of regulatory signaling networks [11]. In bacteria, tandem gene duplications are known to contribute to specific environmental adaptations [39]. Our present findings clearly show that genetic redundancy of *hcpG* and *hcpC* contributes significantly to the mutational robustness of *H. pylori* growth specifically during infection of cultured AGS cells (**Figs. 3a–3f**). Genetic redundancy among paralogs generally tends to be condition-dependent [40]; thus, that effects of *hcpG* and *hcpC* deletions on *H. pylori* growth and fitness were more apparent specifically during infection, a physiologically more relevant condition, and not in pure broth culture is not surprising (**Figs. S5a–S5f in File S1**). What is surprising, however, is that the nature of this genetic redundancy switches from complete to quantitative depending on the temporal context of the infection (**Fig. 3g**). This suggests that *hcpG* and *hcpC* are also coupled via infection-induced regulatory links that mediate such switches. Constituting such a regulatory module can be advantageous for *H. pylori* because depending on when *hcpG* and *hcpC* are active, regulation of distinct processes mediated by them (see discussion below) can be coupled or uncoupled from each other in response to temporal or spatial context of the infection.

The dependence of paralogous redundancy on the context *of H. pylori* infection observed in the present study and in other earlier studies [40], [41] argues against a predominantly compensatory (backup) function of duplicate genes. During early infection, both *hcpG* and *hcpC* are essential for optimal HspB translocation, and neither of them functionally compensates for a lack of their redundant partner. This suggests that *hcpG* and *hcpC* are *specialized* in distinct manners to perform HspB translocation during early infection. This is intriguing, because from middle to late infection, HcpC alone appears to be necessary and sufficient for HspB translocation, whereas HcpG is dispensable and unable to functionally rescue HcpC deletion despite its apparent ability to mediate HspB translocation in early infection (**Fig. 4b–4e**). We ascribe the lack of generic backup functional capacity between *hcpG* and *hcpC* to two distinct factors: dosage amplification and functional or regulatory divergence.

Theoretical studies have suggested that duplicate genes whose products mediate stress responses or generally mediate organism-environment interactions can be retained in genomes of such organisms by positive selection for increased dosage [42], [43]. Specifically, surface-associated HspB appears to be important for gastric colonization early in *H. pylori* infection [44]. Thus, efficient, rapid HspB translocation early during infection should favor successful *H. pylori* colonization. We also observed that during early infection, both *hcpG* and *hcpC* were expressed at relatively low levels in WT HpG27 strain, and expression of both genes was not significantly altered when their redundant partner was deleted (**Fig. 6c**). Thus, given their relatively low expression levels, HcpG and HcpC appear independently selected because of their combined contribution to efficient and rapid HspB translocation in early *H. pylori* infection.

Temporal variation in the relative necessity of *H. pylori* paralogs for HspB translocation indicates the potential regulatory influence of infection-induced cellular signals on HcpG and HcpC activity and function. This regulatory influence is also suggested by our observation that *hcpG* could not functionally rescue *hcpC* deletion (**Figure 4b–4e**). Thus, we speculate that whereas early infection-induced signals likely activate both HcpG and HcpC the transition from early infection to middle to late infection phase predominantly elicits HcpC-activating signals (**Fig. 4f**). Two additional lines of evidence suggest functional divergence between *hcpG* and *hcpC* during late infection: (1) the additive effect of *hcpG* deletion on the *ΔhcpC* phenotype during late infection suggests that *hcpG* and *hcpC* likely perform distinct functions; and (2) positive (or diversifying) selection on extant *hcpG* and *hcpC* allele also suggests potential functional divergence between HcpG and HcpC. Collectively, these data suggest that during late infection *hcpG* and *hcpC* appear to be selected primarily for their divergent functions that are likely regulated by infection-induced signals.

### Evolution of *hcpG* and *hcpC* Duplication in *H. pylori* Populations

**Figure 7:**
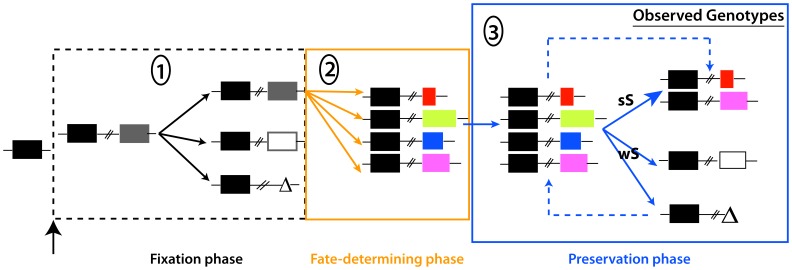
Simplified model of stable maintenance of *hcpC* and *hcpG in H. pylori* populations. The fate of a duplicate gene can be studied in three distinct phases beginning with fixation of the duplicate in the *H. pylori* population [42]. We propose that in the preduplication phase, *hcpC* is fixed in *H. pylori* populations; the fixation phase begins with the origin of *hcpG*. During the fixation phase, the duplicate copy escapes loss because of genetic drift and is fixed. This initiates the second phase, in which the duplicate accumulates fate-determining mutations. We suggest that multiple fate-determining mutations give rise to polymorphic *hcpG* variants. In the preservation phase, *hcpC* and *hcpG* are maintained subject to selection intensity for 1) dosage amplification in early infection or 2) functional divergence during late infection. Strains harboring pseudogenized *hcpG* or lacking *hcpG* altogether are postulated to reflect weak selection or competition among variants for occupation of specific biochemical niches, respectively. The dashed line in the preservation phase indicates cyclical duplication and loss events that may periodically give rise to *hcpG* alleles with new functional capacities; only well adapted *hcpG* variants survive whereas the others are pseudogenized. Black rectangles, *hcpC*; grey rectangle, duplicate copy; white rectangle, pseudogenized *hcpG*; **Δ**, *hcpG* deletion; colored rectangles, *hcpG* variants; sS, strong selection; wS, weak selection.

The context-variable redundancy of *hcpG* and *hcpC* described here may also have broader implications on understanding the evolution of gene duplications. Models explaining the maintenance of paralogs typically invoke functional subdivision and/or novelty in duplicate copies and are classified into multiple categories [42]. Although, our present data best fit the positive dosage model [42], [43], in which paralogs are selected independently because of their cumulative contribution to the same function, additional models may be required because of different redundancy dynamics during late infection. The positive dosage model predicts two possible outcomes based on the strength of selection on cumulative *hcpG* and *hcpC* action: under strong selection, the duplicate copy (*hcpG*) may be quickly fixed whereas under weak selection, a null mutation may become fixed by random genetic drift resulting in *hcpG* pseudogenization, and eventually loss of *hcpG* from *H. pylori* populations (**Fig. 7**). In highly variable environments, such as those in *H. pylori’s* more than three billion human hosts, the strength of selection on increased dosage may periodically wax and wane, leading to cyclical gene duplication and gene loss [45]. Such variation in strength of selection may underlie the three distinct *hcpG* and *hcpC* genotype combinations we observed in *H. pylori* populations (**Fig. 7**). Thus, each strain-specific *hcpG-hcpC* genotype may reflect an individual host-specific adaptation of that *H. pylori* strain. Alternatively, if cyclical bursts of gene duplication and pseudogenization are common then the presence of pseudogenized *hcpG* alleles in *H. pylori* populations could be re-interpreted to reflect inferior *hcpG* variants outcompeted by *hcpG* alleles better adapted to a specific biochemical niche [45]. Such cyclical duplication-pseudogenization may permit selection to explore a wide mutational landscape, likely fixing only those *hcpG* variants that provide additional functional capability (**Fig. 7**). The extreme genetic heterogeneity among functional *hcpG* alleles driven by positive selection further supports the possibility that *hcpG* alleles may encode functionally divergent proteins. Taken together, we propose that context-variable redundant behavior and coupling of paralogs via regulatory links generated by infection-induced signals may have wide-ranging implications on understanding of the evolution of gene duplications, and may require additional sub classification of existing models.

### Implications for *H. pylori* Pathogenesis

Our study has identified two new potential bacterial determinants, HcpG and HcpC, which may contribute to *H. pylori* pathogenesis via regulation of HspB/GroEL/Hsp60 translocation or export to the bacterial surface. The *H. pylori* HspB/GroEL/Hsp60 appears essential for *H. pylori* colonization during early infection [44], for induction of innate immune responses [37], and can enhance angiogenesis [38] and tumorigenesis [46]. In most bacteria the HspB chaperone protein is cytoplasmic but in *H. pylori* this protein is often found on bacterial surface and in the extracellular milieu. The mechanism of HspB export is somewhat controversial. While Phadnis *et al* [35] argued that HspB is reabsorbed to intact cell membranes following its release into the extracellular milieu via autolysis, Vanet and Labigne [36] showed that HspB/GroEL/Hsp60 more likely underwent active secretion rather than autolysis. Because of the apparent HcpG- and HcpC-dependent modulation of HspB translocation in intact unpermeabilized *H. pylori* cells, and the demonstration that HcpC can directly interact with HspB, we favor the idea that HspB may be actively secreted rather than exported via autolysis. The precise mechanism of how *HcpC* and *HcpG* might mediate HspB export, however, remains to be determined. It will be important to determine whether HcpG can also interact directly with HspB, whether HcpG and HcpC co-localize with HspB to the bacterial surface, and identify the infection-induced signals that seem to temporally regulate the functions and/or activity of HcpC and HcpG in the context of infection.

## Materials and Methods

### *H. Pylori* Strains

The 166 H. pylori isolates included in this study were obtained from phylogenetically distinct European (Spain), African (The Gambia and South Africa), East Asian (Japan, South Korea), South Asian (India), and South American (Lima and Shimaa, Peru) populations (**Figure S1 and Table S1 in File S1**). All of these strains were obtained from patients who had sought medical attention and undergone endoscopic biopsies after giving their informed consent at their respective institutions. Detailed listing of strains, along with their culture, growth and maintenance conditions is described in supplementary methods available **in File S1**.

### DNA Extractions, PCRs and Nucleotide Sequencing

Standard procedures were used for extracting *H. pylori* genomic DNA [47], PCR and nucleotide sequencing. Briefly, specific PCRs were carried out in 25 µl reaction mixtures in a PCR buffer supplied by the manufacturer (Biolase; MidSci, St. Louis, MO) and containing 5–10 ng of genomic DNA, 1 U of Taq polymerase (Biolase; MidSci), 1.5 mM MgCl_2_, 0.8 pmol each of forward and reverse primers, and 100 µM dNTP mix for 30–35 cycles of denaturation (94°C), annealing (55°–64°C, as required), and elongation (68°–72°C, as required). PCR products were purified (QIAquick PCR purification kit; Qiagen, Valencia, CA) and then quantified using the Biophotometer (Eppendorf, Hauppauge, NY). Nucleotide sequencing was performed using both DNA strands at the high-throughput genomics unit at the University of Washington, Seattle, WA. The primers used in PCR, and nucleotide sequencing are listed in **Table S2 in File S1**.

### Phylogenetic Analyses and Computations

*hcpC* and *hcpG* nucleotide sequences were assembled and edited using the Seqman suite in the Lasergene software program (DNASTAR, Madison, WI). An *hcpC* multiple sequence alignment (MSA) was generated using MEGA version 5 [48] (www.megasoftware.net). The *hcpG* MSA was generated as follows: an initial MSA was generated by aligning HcpG sequences with crystal structures of *H. pylori* Slr proteins HcpB [49] and HcpC [31] using EXPRESSO [50] (http://igs-server.cnrs-mrs.fr/Tcoffee/tcoffee_cgi/index.cgi). Because of high polymorphism levels in *hcpG* sequences, this alignment was further edited manually based on the biochemical features of amino-acids to correct for obvious mismatches. The resulting MSA was used to manually derive a corresponding nucleotide alignment using MEGA alignment editor. Phylogenetic reconstruction and analyses of selection pressures acting on *hcpC* and *hcpG* codons and lineages were performed using maximum likelihood (ML) methods implemented in PAUP* version 4b10 and PAML version 4.3b, respectively. Best-fit models of DNA sequence evolution used in phylogenetic reconstruction were selected using MODELTEST (**Table S3** and **Table S4 in File S1**). Details of these analyses are described in supplementary methods available **in File S1**. Phylogenetic datasets generated in this study have been submitted to GenBank® (Accession numbers: 1) *hcpC* dataset, KC007946–KC008026 and 2) *hcpG* dataset KC008027–KC008064).

#### Population genetic computations

Estimates of total nucleotide diversity (π), Waterson’s θ, nucleotide diversity at synonymous and nonsynonymous sites (π_S_ and π_A_), genetic differentiation among populations (F_ST_) with accompanying permutation tests, and the McDonald-Kreitman tests and associated estimates of α, the proportion of amino acids under positive selection [51] were obtained using the DNASP software program (version 5.1) [52].

#### Structural and domain architecture analyses

Domain architecture analysis of *hcpG* and *hcpC* sequences was performed using the Simple Modular Architecture Research Tool (SMART) [53]. Positively selected residues of HcpC were mapped to the surface of the HcpC crystal structure (PDB code 1OUV [31]) using the PYMOL molecular visualization system (http://www.pymol.org).

### *H. pylori* Genetic Engineering

*hcpC* and *hcpG* knockout derivatives *(ΔhcpC* and *ΔhcpG* single mutants and *ΔhcpC*,*ΔhcpG* double mutant) of *H. pylori* strain G27MA and the *hcpG*::6xHIS knock-in G27MA strain were generated using the streptomycin contraselection-based method described previously [54] while incorporating small modifications. Strategy for generating knockout and knock-in strains is shown in **Fig. S2** in **File S1** and is described in detail in supplementary material available in **File S1**.

### Growth and Fitness Assays

#### *In vitro* growth and fitness assays

*H. pylori* G27MA WT and its *hcpG* and/or *hcpC* deletion derivatives were grown on BHI plates containing appropriate selective antibiotics for 3 days. Fifty milliliter tissue culture flasks were then inoculated with a bacterial suspension derived from plate cultures (OD_600_ = 0.05/mL for each strain). A liquid medium comprising BHI broth supplemented with 1% IsoVitaleX, 1% *H. pylori*-selective supplement, and 10% fetal bovine serum (FBS) (GIBCO, CA) was used to culture the *H. pylori* strains. Flasks were initially incubated at 37°C for 30 min in a 5% CO_2_ incubator and then transferred to GasPak jars and incubated at 37°C with shaking (120 rpm) for a maximum of 56 h [55]. At specific intervals, cell aliquots were from culture flasks were diluted serially and plated on selective BHI plates to enumerate the WT and mutant colony-forming units (CFUs). The log-transformed CFU mL^−1^ count was used to determine the competitive index (CI) in co-culture experiments. The CI was calculated according to the ratio of mutant to WT bacteria at each time point compared with the ratio of mutant to WT bacteria in the inoculum [56], [57]. A CI value greater than one indicated that the mutant out-competed the WT, whereas a value less than or equal to one indicated that the WT out-competed the mutant. Growth assays in pure cultures and fitness assays in broth co-cultures were repeated three times, and the statistical significance of observed differences in the growth or fitness of *hcpC* and/or *hcpG* mutants and WT G27MA was determined using a t-test with α = 0.05.

#### Growth and fitness assays during *H. pylori* infection of cultured AGS cells

Before each experiment in cultured AGS cells, bacteria were passaged once on BHI horse blood agar plates under standard microaerobic conditions as recommended [33]. AGS cells (ATCC CRL 1739) were normally cultured and maintained in antibiotic-free high-glucose Dulbecco’s modified Eagle’s medium (DMEM) supplemented with 10% heat-inactivated FBS. Cells were allowed to grow and maintained in 50 mL tissue culture flasks at 37°C in a humidified atmosphere of 5% CO_2_. AGS cells were seeded at a density of 1×10^5^ cells/mL into six-well plates and then allowed to grow to 80% confluence [58]. Just prior to infection of cultured AGS cells with *H. pylori*, the cell-medium mixture was removed and replaced with fresh DMEM containing 20% FBS. Bacteria were harvested from pure liquid cultures in BHI and washed in phosphate-buffered saline (PBS; pH 7.4); AGS cells were infected at an MOI of ∼ 100 *H. pylori* cells per AGS cell. Plates were centrifuged for 10 min at 1,000 g to ensure bacterial contact with the AGS cells and incubated at 37°C in a humidified atmosphere of 5% CO_2_. At specific intervals cells were gently scraped from a well, mixed, diluted serially, and plated on selective BHI plates to enumerate the WT and mutant CFUs. In co-cultures experiments the CI was calculated as described above. Each experiment was repeated five more times, and the statistical significance of observed differences in WT and mutant strains was calculated using a *t-*test with α = 0.05.

### FACS, Immunoblot Analysis and ELISA

#### Antibodies

Antibodies used in this study are listed in supplementary methods available in **File S1**.

#### HspB expression dynamics in vitro and during infection

HspB expression was studied in unpermeabilized bacterial cells grown in pure cultures and during infection of cultured AGS cell using a FACS-Calibur™ flow cytometer (BD, Franklin Lakes, NJ). The data were analyzed using the WinMDI software program (version 2.9). HspB, CagA and activated-Mitogen Activated Protein Kinase (MAPK, ERK2) expression during infection was studied using serum-deprived AGS cells that were allowed to grow to 80% confluence. Parameters used in the FACS analyses are listed in **Table S5** in **File S1**. Detailed methods for FACS analyses are presented in supplementary material available in **File S1**.

#### Protein expression, purification and immunoassays

HcpC fused to N-terminal MBP and C-terminal histidine tags was expressed and purified as described previously [26]. HspB (gene *hp0010* from *H. pylori* strain 26695) was cloned into a pGEX-6P expression vector (GE Healthcare) and purified GST-HspB was concentrated using ultrafiltration and stored in PBS buffer supplemented with 10% (v/v) glycerol at −80°C. All ELISA experiments were performed using Nunc Maxisorp 96-well plates at volumes of 100 µL/well. Details of HspB expression, purification and subsequent use in ELISA are provided in supplementary methods available in **File S1**.

### Reverse-transcription and Real-time PCRs

Standard methods were used and are detailed in supplementary material available in **File S1**.

## Supporting Information

File S1 Detailed description of methods and supplementary figures (Fig. S1– Fig. S6) and tables (Table S1–Table S10).
(DOCX)
