# Supplementary material for: Robustness of Helicobacter pylori Infection Conferred by Context-Variable Redundancy among Cysteine-Rich Paralogs
Source: PLoS One. 2013 Mar 26;8(3):e59560. doi: 10.1371/journal.pone.0059560 (PMC3608669; doi:10.1371/journal.pone.0059560)
Supplement: File S1 — Detailed description of methods and supplementary figures (Fig. S1– Fig. S6) and tables (Table S1–Table S10). (DOCX) [file pone.0059560.s001.docx]

**Robustness of *Helicobacter pylori* Infection Conferred by Context-Variable Redundancy among Cysteine-Rich Paralogs**

Kalyani Putty^1^, Sarah A. Marcus^1*^, Peer R.E. Mittl^3^, Lindsey E. Bogadi^1^, Allison M. Hunter^1^, Swathi Arur^5^, Douglas E. Berg^4^, Palaniappan Sethu^2^, and Awdhesh Kalia^1, 6¶^

**Supplementary information**

**Supplementary Detailed Methods.**

**Supplementary Figures, Fig. S1 – Fig. S6**

**Supplementary Tables, Table S1 – Table S10.**

**SUPPLEMENTARY DETAILED METHODS**

***H.pylori* strains, culture and growth conditions.**

All strains included in this study were described earlier [[1](#_ENREF_1),[2](#_ENREF_2),[3](#_ENREF_3),[4](#_ENREF_4),[5](#_ENREF_5),[6](#_ENREF_6)]. The bacteria were grown on brain heart infusion (BHI) agar (Difco, Lawrence, KS) supplemented with 7% defibrinated horse blood (Cleveland Scientific, Bath, OH), BBL medium enrichment for fastidious microorganisms (IsoVitalex; Becton Dickinson, France), *H. pylori* selective supplement (Dent; 10 mg/L vancomycin, 5 mg/L cefsulodin, 2,500 U/L polymyxin B, 5 mg/L trimethoprim and 7.5 mg/L amphotericin B) (Oxoid Ltd, Basingstoke, Hants, England). Erythromycin (15 μg/mL), streptomycin (10 μg/mL), and/ or chloramphenicol (15 μg/mL) was added to BHI-agar-containing horse blood and IsoVitalex as needed to select *hcpG* and *hcpC* mutant colonies. Bacterial cultures were typically incubated at 37°C for 3-4 days in a GasPak jar (BBL Microbiology Systems, Cockeysville, MD) with a microaerobic gas mixture (CampyPak, BBL Microbiology Systems) composed of 5% oxygen, 10% carbon dioxide, and 85% nitrogen. The strains were maintained as frozen stocks in BHI broth containing 15% glycerol and stored at -74°C until required.

***Phylogenetic reconstructions****.*

Phylogenetic trees based on *hcpC* and *hcpG* MSAs were generated using the ML optimality criterion as implemented in PAUP* [[7](#_ENREF_7)] essentially as described previously [[4](#_ENREF_4),[5](#_ENREF_5)]. Briefly, an initial neighbor-joining tree was generated using BIONJ and then used as an input tree to select the best fit evolutionary model using MODELTEST [[8](#_ENREF_8)] **(supplementary Tables S2 and S3)**. ML trees were reconstructed via heuristic searches using the TBR algorithm implemented in PAUP*. The significance of the observed grouping in the phylogenetic trees was determined using 1000 bootstraps conducted according to the neighbor-joining criterion while incorporating the ML-optimized substitution matrix parameters. Trees were viewed and edited using the MEGA Tree Explorer.

***Analysis of selection pressures.***

Selective pressures acting on *hcpC* and *hcpG* codons and lineages were measured using the CodeML application implemented in PAML version 4.4e [[9](#_ENREF_9)]. CodeML uses an ML method that takes into account the sequence phylogeny and assesses the data fit of various models of codon evolution [[10](#_ENREF_10)] that differ in how ω (rate of nonsynonymous changes/rate of synonymous changes=d_N_/d_S_) varies across the sequence or phylogeny. ML-optimized *hcpC* or *hcpG* phylogenies were used as input for CodeML analysis after converting the branch lengths to nt substitutions per codon from nt substitutions per site. Site-specific models in codon-based analysis assume a single ω for all branches of the input tree, but allow ω to vary among individual codon sites. Thus, such models provide a measure of heterogeneity in selection pressures acting across gene sequences. The following codon substitution models were used: M0 (One-ratio), M1a (Neutral model), M2a (selection), M3 (discrete), M7, M8 and M8a. Positive selection was inferred when codons with ω_­S_ > 1 were identified and the likelihood score (-InL) of the codon substitution model was significantly higher than that of a nested model that did not take positive selection into account. For detecting positive selection in *hcpC* or *hcpG* codons three sets of model comparisons were used as recommended [[11](#_ENREF_11)]: M1a versus M2a, M7 versus M8, and M8 versus M8a. The M1a-M2a model comparison is considered more robust (or less powerful) in detecting positive selection than is the M7-M8 comparison [[11](#_ENREF_11)]. The M8a-M8 comparison tests the null hypothesis of neutral evolution by fixing ω at 1 in the M8a model, whereas the M8 model estimates ω freely according to the sequence data [[12](#_ENREF_12)]. The probability that a specific codon belonged to the neutral, negative, or positively selected class was calculated using the Bayes-Empirical-Bayes method [[11](#_ENREF_11)] implemented in PAML. Multiple runs of CodeML, assuming different initial ω and κ values and different models for estimating equilibrium codon frequencies (calculated according to the average nt frequencies at the three codon positions tables (F3X4) or used as free parameters) were analyzed for *hcpC and hcpG* to verify the convergence optima for each model. Finally, to measure the rate-variation in selection along *hcpC* or *hcpG* phylogeny, the free-ratios codon-substitution model (M1bra) implemented in PAML was used. M1bra estimates the ω-ratio for each branch in the phylogenetic tree, and its fit to the data is determined via comparison with the M0 model, which assumes a single rate of codon evolution for the given phylogeny.

***H. pylori Genetic Engineering***

*Generation of ΔhcpC and ΔhcpG mutant derivatives. hcpC* and *hcpG* knockout derivatives of *H. pylori* strain G27MA were generated using small modifications of the streptomycin contraselection-based method described previously [[13](#_ENREF_13)]. The overall specific strategies for generation of insertion and deletion *hcpG* and/or *hcpC* alleles are summarized in **supplementary Fig. S2**. Briefly, streptomycin-resistant (*rpsL*-mutant) derivatives of the cell culture-adapted *H. pylori* strain GM27MA [[13](#_ENREF_13)] were grown on supplemented BHI agar (as above) under standard microaerobic conditions (5% O_2_, 10% CO_2_) at 37°C. Insertion and deletion alleles were prepared using PCR products A, B and C, which were generated with primer numbers 1-6 listed in **supplementary** **Table S2**. These three PCR products were assembled into one DNA using different strategies for *hcpC* and *hcpG*. For *hcpC* deletion, two sequential assembly PCRs were performed to generate fragment AB with primers 1 and 6 and generate fragment BC with primers 4 and 5 (**supplementary** **Fig. S2a**). The AB and BC fragments were stitched together with primers 1 and 4 to generate the ABC knockout assembly. The initial *rpsL,erm* (streptomycin-susceptible, erythromycin-resistant) replacement alleles were generated using *H. pylori* strain 26695 genomic DNA and recovered in the Str^R^ 26695 derivative, and then moved to Str^R^ GM27MA via DNA transformation. However, this strategy failed to provide good yields of the ABC assembly for generating the *hcpG* deletion. Thus, the strategy used for *hcpC* was slightly modified. Specifically, EcoRI and NotI restriction sites were introduced at the 5’ termini of fragments A and C and the 5’ and 3’ ends of the *rpsL,cat* cassette [[14](#_ENREF_14)] (**supplementary** **Fig. S2b**). The ABC knockout assembly was generated via ligation of the restricted fragments and amplified by PCR with primers 1 and 4. This strategy provided the most consistent results in generating the “ABC” knockout or knock-in assemblies (**supplementary** **Fig. S2c**).

For natural transformation, culture of an *rpsL*-mutant (Str^R^) *H. pylori* strain freshly grown overnight was streaked on BHI agar and incubated for 4 h. One microgram of a PCR product or genomic DNA containing the *rpsL,ery or rpsL,cat*  cassette at the desired chromosomal locus was added in a 1 cm diameter spot. The culture with the added DNA was incubated overnight and the resultant bacterial growth was streaked on fresh BHI agar containing erythromycin or chloramphenicol to select for transformant colonies. Isolated single Ery^R^ or Cat^R^ colonies appeared on BHI plates after 48-72 h incubation and were picked with sterile toothpicks to inoculate BHI streptomycin (10 µg /mL) and BHI erythromycin or BHI chloramphenicol. Ery^R^ or Cat^R^ colonies that were Str^S^ were identified, and replacement of the targeted gene (*hcpC* or *hcpG*) with the *rpsL,ery or rpsL,cat* cassette, respectively, in several such transformants was verified using PCR, as described previously [[13](#_ENREF_13)].

*Generation of the ΔhcpC,ΔhcpG double mutant*. To generate the *ΔhcpC,ΔhcpG* double mutant, genomic DNA was extracted from the HpG27MA*ΔhcpC* strain and 10 μL of this DNA was used to transform the G27MA*ΔhcpG* Cat^R^ strain as described above. The resulting bacterial growth was streaked on fresh BHI erythromycin-chloramphenicol plates. After growing for 3-4 days, individual Erm^R^,Cat^R^ colonies were picked using sterile toothpicks and streaked on BHI chloramphenicol-erythromycin and BHI-streptomycin plates, respectively. Erm^R^,Cat^R^ colonies that were Str^S^ then had their genomes amplified via PCR using primers 1 and 4 (**supplementary** **Table S2**) to verify the stable integration of replacement cassettes in the *ΔhcpC,ΔhcpG* double knockout.

*Knock-in of the hcpG::6Xhis assembly into the G27MAΔhcpG strain*. The strategy used to generate the *hcpG* knockout assembly was used for replacing the *rpsL,catR* cassette in the G27MA*ΔhcpG* derivative with a *hcpG::*6X*his* fusion assembly (fragment B’) generated using PCR with primers 5 and 6 (**supplementary Fig. S2c**; **supplementary** **Table S2**). The resulting AB’C assembly was used to transform the *H. pylori* G27MA*ΔhcpG* strain as described above. The transformants were inoculated on to BHI-streptomycin and BHI-chloramphenicol plates, and colonies determined to be Str^R^ and Cat^S^ were tested using PCR to confirm the presence of the *hcpG::6Xhis* fusion construct.

**FACS, Immunoblot analysis and ELISA**

*Antibodies used in this study*: The anti-CagA (goat IgG) sc-34039 and anti-*H. pylori* HSP (mouse IgG) sc-57779 antibodies were obtained from Santa Cruz Biotechnology (Santa Cruz, CA). Anti-phosphorylated-MAPK (α-MAPKYT; rabbit IgG) M-76783, anti-His (mouse IgG), and anti-tubulin (mouse IgG) antibodies were obtained from Sigma (St. Louis, MO). Secondary antibodies used in this study were the following: horseradish peroxidase (HRP)-conjugated secondary antibodies (HRP-mouse and HRP-goat), goat polyclonal secondary antibody to mouse IgG - H&L (fluorescein isothiocyanate [FITC]) ab6785, donkey polyclonal secondary antibody to goat IgG - H&L (FITC) ab6881, donkey F(ab')2 polyclonal secondary antibody to rabbit IgG - H&L (phycoerythrin [PE]) ab7007, goat polyclonal secondary antibody to mouse IgG - H&L (Texas red) ab6787 were obtained from Abcam (Cambridge, MA).

*HspB expression dynamics during in vitro growth*. Pure *H. pylori* cultures were grown in BHI broth as described above. At specific intervals, 3 mL aliquots of bacterial culture were, centrifuged at 3500 rpm for 10 min, and washed twice with 1x PBS (pH 7.4). Bacterial cells were immediately fixed in 4% paraformaldehyde at room temperature for 15 min, and then washed twice with 1x PBS and stored at 4^0^C. For staining with anti-HSP the cell permeabilization step was excluded to specifically stain the bacterial cells for surface HspB. An anti-HSP primary antibody was added to fixed, unpermeabilized bacterial cells to a final concentration of 1 μg/mL, and incubated on ice for 30 min followed by washing twice with 1x PBS containing 0.2% bovine serum albumin (BSA) (B-PBS). A secondary antibody conjugated to a Texas Red fluorophore was then added to this cell suspension at a final concentration of 0.5 μg/mL and incubated for 30 min on ice in the dark. Cells were then washed twice with B-PBS. Cells were analyzed using a FACS-Calibur^TM^ flow cytometer (BD, Franklin Lakes, NJ) with channel FL4 to detect the fluorescence emitted from 10^4^ cells/sample. Unstained bacteria were used to optimize the scatter (forward and side scatter) and establish back ground fluorescence. After determining the mean fluorescence in each sample, the data were analyzed using the WinMDI software program (version 2.9) to calculate the mean geometric fluorescence values and the fluorescent intensities were depicted as histograms. Parameters used in the FACS analyses are listed in **supplementary** **Table S4**.

*HspB expression dynamics during H. pylori infection of cultured AGS cells***.** Infection of serum-deprived AGS cells with *H. pylori* strains was performed essentially as described previously [[15](#_ENREF_15),[16](#_ENREF_16)]. Briefly, 48 h before infection, AGS cells were seeded in six-well plates at a density of 0.5 x 10^5^ cells per well and allowed to grow to 80% confluence. Twelve h before infection, the cell-medium was replaced with fresh serum-free medium. Before each experiment, bacteria were passaged once on BHI horse blood agar plates under standard microaerobic conditions. Serum-deprived AGS cells were infected at an MOI of about 100 bacteria per AGS cell. Plates were centrifuged for 10 min at 1,000g to ensure bacterial contact with the AGS cells, and incubated at 37°C in a humidified atmosphere of 5% CO_2_. At specific intervals AGS cells were washed twice with 1x PBS to remove unattached bacteria and detached using 2 mM EDTA. Collected cells were immediately fixed in 4% paraformaldehyde at room temperature for 15 minutes. Staining of unpermeabilized, fixed cells for HspB surface expression was performed as described above. Unstained AGS cells with adherent bacteria were used to optimize the forward and side scatter and establish the background fluorescence. Uninfected AGS cells were used as negative controls. The experiment was performed three times, and a *t*-test was used to calculate statistical significance of observed differences in HspB fluorescence in the experimental groups.

*CagA and activated MAPK expression analyses during infection***.** CagA is a type IV effector protein that is translocated into host cells following contact of *H. pylori* with mammalian cells; this translocation results in activation of cellular MAPK [[17](#_ENREF_17)]. These signaling events are considered hallmarks of *H. pylori* infection. To determine whether *hcpC* and/or *hcpG* deletion generally affected infection-induced signaling events during *H. pylori* infection of cultured AGS cells CagA and activated MAPK expression along with HspB expression were monitored. For this purpose, at specific intervals after infection, infected AGS cells were collected and fixed immediately in 4% paraformaldehyde. Because both CagA and MAPK are intracellular, the cells were permeabilized with 0.01 Triton-X 100 for 3 min at room temperature. Anti-CagA and anti-MAPKYT antibodies were added to the permeabilized cell suspension to a final concentration of 1 μg/mL and incubated on ice for 30 min followed by two washes in B-PBS. Secondary antibodies conjugated with either PE (for anti-activated MAPK) or FITC (for anti-CagA) fluorophores were added to the cell suspension at a final concentration of 0.5 μg/mL and incubated for 30 minutes on ice in the dark. Cells were then washed twice with B-PBS and analyzed using the FACS Calibur with channel FL1 (FITC) and/or channel FL2 (PE) to detect emitted fluorescence from 10^4^ cells per sample. Unstained AGS cells with adherent bacteria were used to optimize the scatter and establish background fluorescence. Uninfected AGS cells were used as controls. Dot plots were generated using WinMDI (version 2.9).

*Immunoblotting*. Standard procedures were used for immunoblotting of *H. pylori* infected AGS cells. Primary antibodies against CagA, activated MAPK, tubulin and His-tag were detected using HRP-conjugated secondary antibodies and visualized using Western Lightning Chemiluminescence Reagent (Pierce, Rockford, IL) according to the manufacturer's instructions, with a Versadoc 4000 (Bio-Rad Laboratories, Hercules, CA). The blot membranes were stripped for 30 min at 50°C in stripping buffer (62.5 mM Tris-HCl, pH 6.7, 100 mM 2-mercaptoethanol, 2% SDS) for reprobing the same membrane with different antibodies.

***Protein expression, purification and immunoassays****.*

*HspB expression*. HspB (gene *hp0010* from *H. pylori* strain 26695) was cloned into a pGEX-6P expression vector (GE Healthcare). Proper insertion of the gene into the vector was confirmed via DNA sequencing, revealing that the GST-HspB fusion protein differed from the expected amino acid sequence (Uniprot-ID P42383) by a Thr519Ile mutation. The GST-HspB fusion protein was expressed in *Escherichia coli* strain BL21 in Luria-Bertani medium for 3 h at 20°C following induction with 0.5 mM isopropyl β-D-1-thiogalactopyranoside.

*GST-HspB purification*: Induced bacterial cells were harvested by centrifugation, re-suspended in PBS buffer (140 mM sodium chloride, 20 mM sodium phosphate, pH 7.1) and lysed using a French-pressure cell press. After clearing the cell lysate using centrifugation, the supernatant was loaded onto a glutathione affinity column (GE Healthcare). The column was washed with 10 column volumes of PBS buffer. The GST-HspB fusion protein was eluted with 5 mM glutathione in PBS buffer. Purified GST-HspB was concentrated using ultrafiltration and stored in PBS buffer supplemented with 10 % (v/v) glycerol at -80°C.

*Immunoassays*. All ELISA experiments were performed using Nunc Maxisorp 96-well plates at volumes of 100 μL/well. ELISA plates were washed three times with water and three times with TBST (0.2% (v/v) Tween-20 in TBS (0.14 M sodium chloride, 10 mM Tris/HCl, pH 7.4)). Wells were coated with MBP-HcpC in TBS (2.5 ng/μL) for 4 h at room temperature. Immobilized BSA was used as a negative control. To prevent nonspecific binding, plates were blocked with 200 μL per well 0.5% (w/v) BSA in TBST (B-TBST) over night at 4°C. Serial dilutions of GST-HspB in B-TBST were added to the wells and incubated for 70 min at room temperature. Bound GST-HspB was detected using a primary anti-GST goat antibody (1:5000 dilution in B-TBST, GE Healthcare) and a secondary anti-goat IgG antibody conjugated to alkaline phosphatase (1:5000 dilution in B-TBST, Sigma). These antibodies were incubated on the plate for 1 h at room temperature. The amounts of bound antibodies were determined using p-nitrophenyl phosphate under standard assay conditions. Absorption at a wavelength of 405 nm was quantified using a Tecan Genios plate reader.

***Reverse transcription and Real time PCR analyses****.*

*RNA extraction and cDNA synthesis.* RNA extractions from pure *H. pylori* cultures or *H. pylori* infected AGS cells were performed using an RNeasy mini kit (Qiagen) following the manufacturer’s instructions. RNA was dissolved in 30 µL of RNase free water and its integrity was determined with the Agilent 2100 Bioanalyzer (Agilent technologies, Santa Clara, CA). RNA preparations with RIN-value[[18](#_ENREF_18)] greater than nine were used for subsequent cDNA synthesis. cDNA was synthesized using the purified total RNA with the QuantiTect reverse transcription kit (Qiagen) as per the manufacturer’s instructions, and used as a template for reverse transcription PCR and Real time (RT)-PCR. cDNA was used immediately in PCR assays or stored at -20^o^C.

*Qualitative reverse transcription PCR*. cDNAs from geographically distinct bacterial strains that were chosen to reflect the diversity of the *hcpG* domain architecture were prepared from either pure bacterial cultures grown in BHI broth for 24 h or cultured AGS cells 6 h after infection. Standard PCR conditions were used to amplify the *hcpG* and control transcripts with the specific PCR primers listed in **Table S2**.

*Quantitative RT-PCRs*. RT-PCRs analysis was performed essentially following recommended guidelines [[19](#_ENREF_19)]. PCR primers used in these analyses (**Table S2**) were designed using the Primer Express software program (version 3.00; Life Technologies Corporation, Carlsbad, CA) to ensure their compatibility with SYBR green assays. PCRs were performed in 20 µL mixtures containing 2 µL of 1:5 diluted cDNA, 1x Power SYBR green PCR master mix (10 µl) (Life Technologies Corporation), 1 µL each of forward and reverse primer (5 µM) and water to make up the remaining volume. RT-PCRs were performed with a Step-One thermocycler (Life Technologies Corporation) under the following PCR conditions: 95^o^C for 10 min followed by 95^o^C for 15 s and 60^o^C for 1 min for 40 cycles. Appropriate non-RT and non-template controls were included in each reaction plate, and melting curve analysis was performed at the end of each run to confirm the specificity of the reaction. Relative quantification of the target transcripts was determined by the 2^-ΔΔC^_T_ method [[19](#_ENREF_19)] and values were normalized according to either *ureA or recA* levels. Negative and positive values were interpreted as fold downregulation or upregulation of expression of the target transcript. One-way analysis of variance was used for statistical analyses.

**References.**

1. Kersulyte D, Mukhopadhyay AK, Velapatino B, Su W, Pan Z, et al. (2000) Differences in Genotypes of Helicobacter pylori from Different Human Populations. J Bacteriol 182: 3210-3218.

2. Mukhopadhyay AK, Kersulyte D, Jeong J-Y, Datta S, Ito Y, et al. (2000) Distinctiveness of Genotypes of Helicobacter pylori in Calcutta, India. J Bacteriol 182: 3219-3227.

3. Aspholm-Hurtig M, Dailide G, Lahmann M, Kalia A, Ilver D, et al. (2004) Functional Adaptation of BabA, the H. pylori ABO Blood Group Antigen Binding Adhesin. Science 305: 519-522.

4. Kalia A, Mukhopadhyay AK, Dailide G, Ito Y, Azuma T, et al. (2004) Evolutionary Dynamics of Insertion Sequences in Helicobacter pylori. J Bacteriol 186: 7508-7520.

5. Ogura M, Perez JC, Mittl PRE, Lee H-K, Dailide G, et al. (2007) *Helicobacter pylori* evolution: lineage- specific adaptations in homologs of eukaryotic Sel1-like genes. PLoS Comput Biol 3: e151.

6. Kersulyte D, Kalia A, Gilman RH, Mendez M, Herrera P, et al. (2010) *Helicobacter pylori* from Peruvian Amerindians: Traces of Human Migrations in Strains from Remote Amazon, and Genome Sequence of an Amerind Strain. PLoS ONE 5: e15076.

7. Swofford DL (2000) PAUP*. Phylogenetic Analysis Using Parsimony (*and Other Methods). . Sunderland, MA: Sinauer Associates.

8. Posada D (2003) Using MODELTEST and PAUP* to select a model of nucleotide substitution. Curr Protoc Bioinformatics Chapter 6: Unit 6 5.

9. Yang Z (2007) PAML 4: Phylogenetic Analysis by Maximum Likelihood. Molecular Biology and Evolution 24: 1586-1591.

10. Yang Z, Nielsen R, Goldman N, Pedersen AM (2000) Codon-substitution models for heterogeneous selection pressure at amino acid sites. Genetics 155: 431-449.

11. Yang Z, Wong WS, Nielsen R (2005) Bayes empirical bayes inference of amino acid sites under positive selection. Mol Biol Evol 22: 1107-1118.

12. Swanson WJ, Nielsen R, Yang Q (2003) Pervasive adaptive evolution in mammalian fertilization proteins. Mol Biol Evol 20: 18-20.

13. Dailidiene D, Dailide G, Kersulyte D, Berg DE (2006) Contraselectable streptomycin susceptibility determinant for genetic manipulation and analysis of Helicobacter pylori. Appl Environ Microbiol 72: 5908-5914.

14. Styer CM, Hansen LM, Cooke CL, Gundersen AM, Choi SS, et al. (2010) Expression of the BabA adhesin during experimental infection with Helicobacter pylori. Infect Immun 78: 1593-1600.

15. Asahi M, Azuma T, Ito S, Ito Y, Suto H, et al. (2000) Helicobacter pylori CagA protein can be tyrosine phosphorylated in gastric epithelial cells. J Exp Med 191: 593-602.

16. Mimuro H, Suzuki T, Tanaka J, Asahi M, Haas R, et al. (2002) Grb2 Is a Key Mediator of Helicobacter pylori CagA Protein Activities. Molecular Cell 10: 745-755.

17. Hatakeyama M, Higashi H (2005) Helicobacter pylori CagA: a new paradigm for bacterial carcinogenesis. Cancer Science 96: 835-843.

18. Schroeder A, Mueller O, Stocker S, Salowsky R, Leiber M, et al. (2006) The RIN: an RNA integrity number for assigning integrity values to RNA measurements. BMC Mol Biol 7: 3.

19. Schmittgen TD, Livak KJ (2008) Analyzing real-time PCR data by the comparative CT method. Nat Protocols 3: 1101-1108.

Supplementary Figures

**
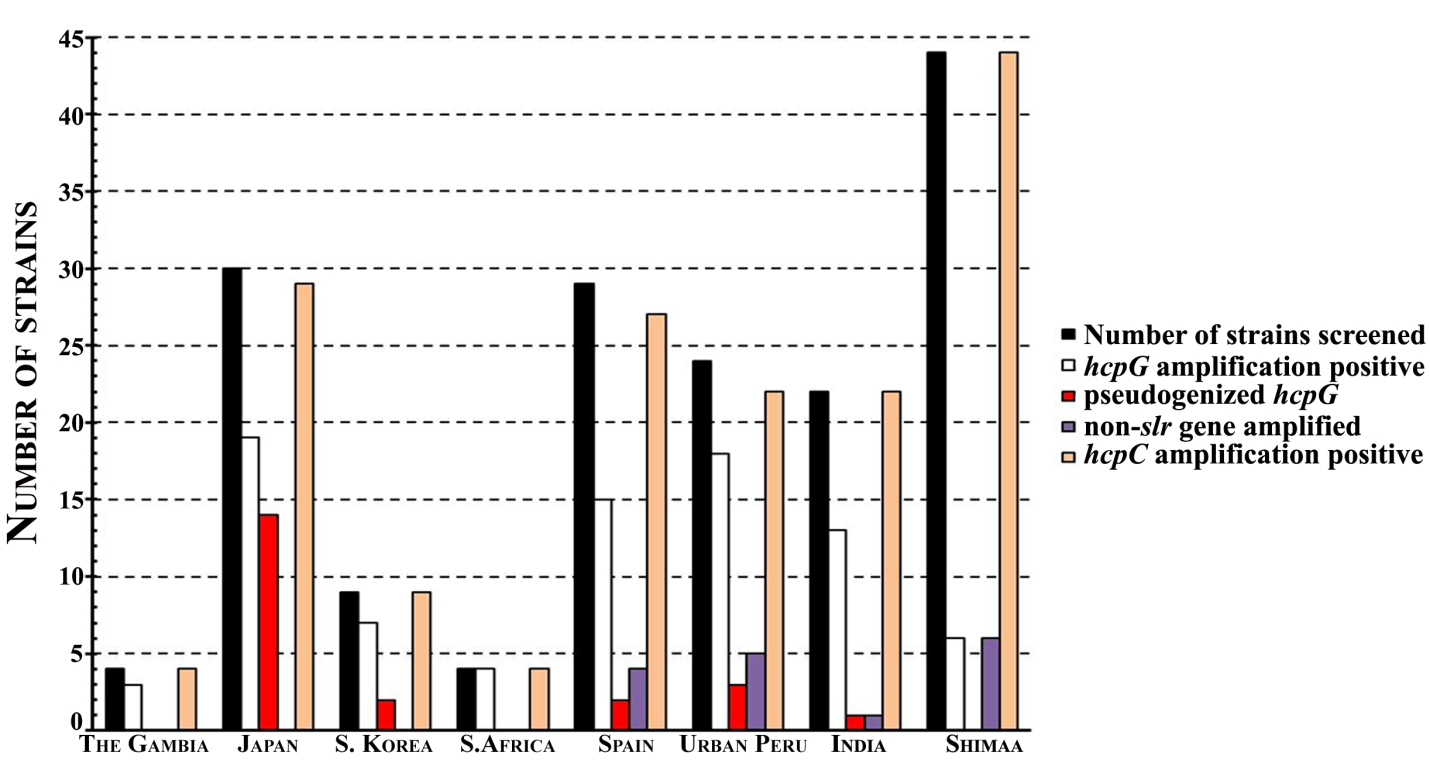
**

Figure S1. Geographic breakdown of *H. pylori* strains included in this study, and PCR –sequencing results for *hcpG* and *hcpC.* Complete listing of strains along with geographic origin is provided in supplementary Table 1A.


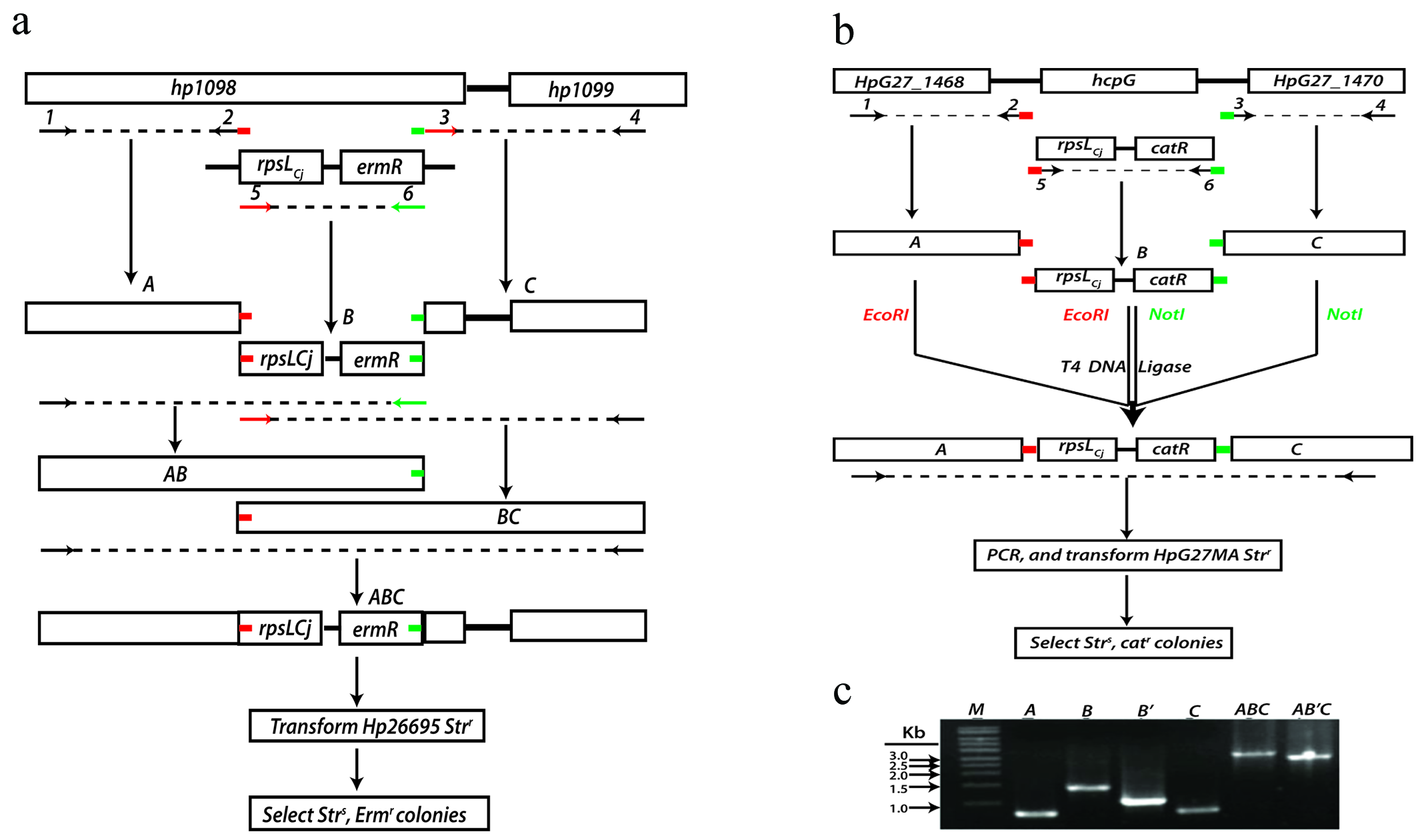


Figure S2. Strategies used for generating mutant derivatives of *H. pylori* G27MA. (a) *hcpC* deletion. The A and C DNAs correspond to chromosomal sequences within and downstream from *hcpC,* respectively. The red and green overhangs in primers 2 and 3 are complementary to and overlap primers 5 and 6, respectively. Fragment B corresponds to the *rpsL,erm* cassette [[13](#_ENREF_13)]. (b) *hcpG* deletion. The red and green 5’- overhangs in the PCR primers correspond to the EcoRI and NotI restriction sites, respectively. Fragments A and C correspond to genes upstream of and downstream from *hcpG*, respectively, whereas fragment B is the *rpsL,cat* cassette. (c) Gel electrophoresis analysis of fragments A, B, B’ (*hcpG::6Xhis*), and C and the ABC and AB’C assemblies used for creating the *ΔhcpG* derivative of G27MA and introducing the *hcpG*::6XHis fusion assembly back into the G27MA*ΔhcpG* strain, respectively.

**
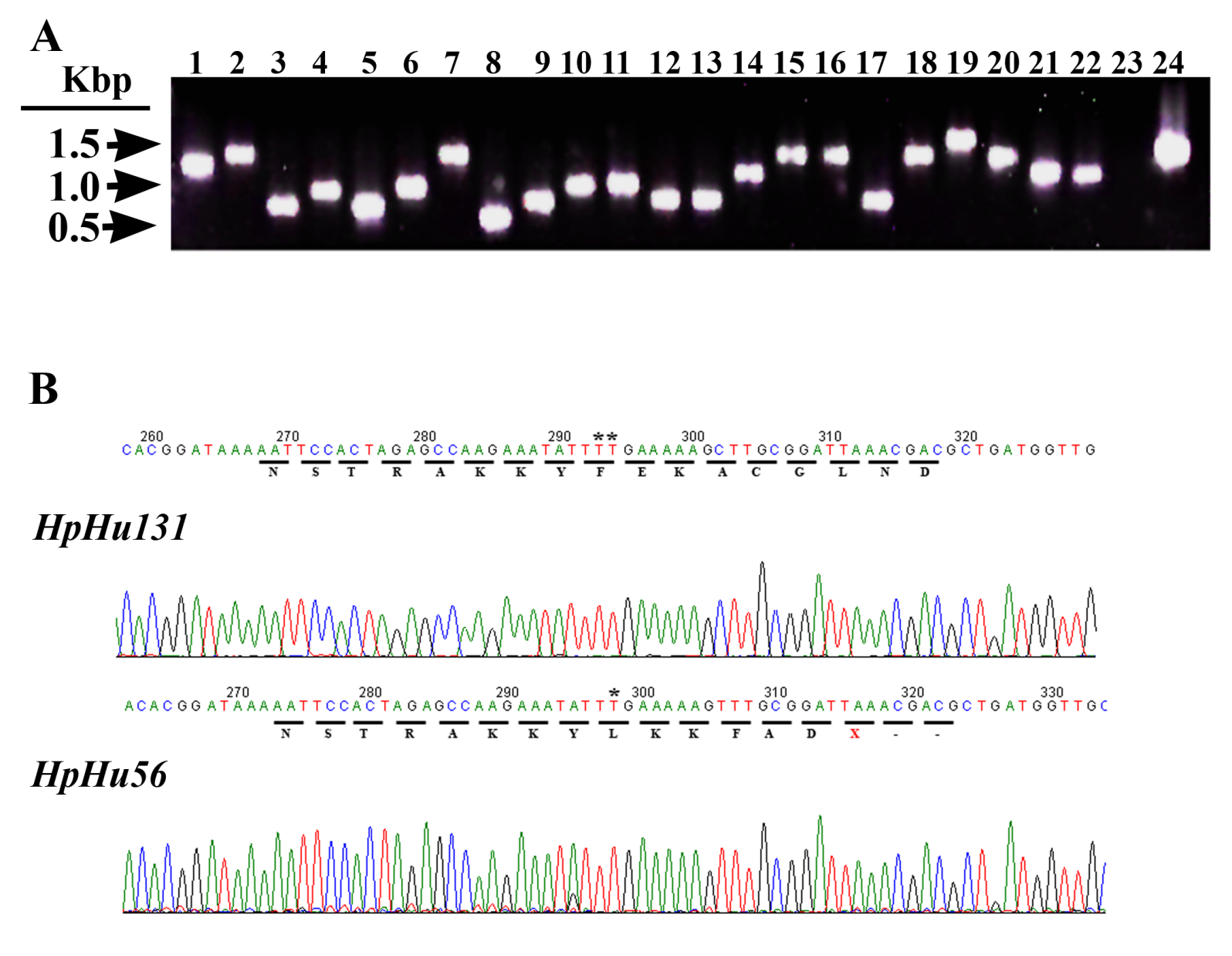
**

**Figure S3. *hcpG* alleles are highly polymorphic and frequently accumulate premature nonsense mutations*.* (a)** Representative agarose gel showing striking size variation in *hcpG* PCR products amplified from a geographically diverse collection of isolates. PCR products from The Gambia (lanes 1-3), Japan (lanes 4-12), South Korea (lanes 13-19), and Spain (lanes 20-22); and lanes 23 and 24 show negative (water) and positive (HpJ99) PCR controls. (**b)** Representative sequence chromatograms from *H. pylori* strains HpHu131 and HpHu56 showing that a frameshift mutation (*ΔT*, indicated by asterisks) in HpHu56 resulted in premature truncation of the encoded protein.


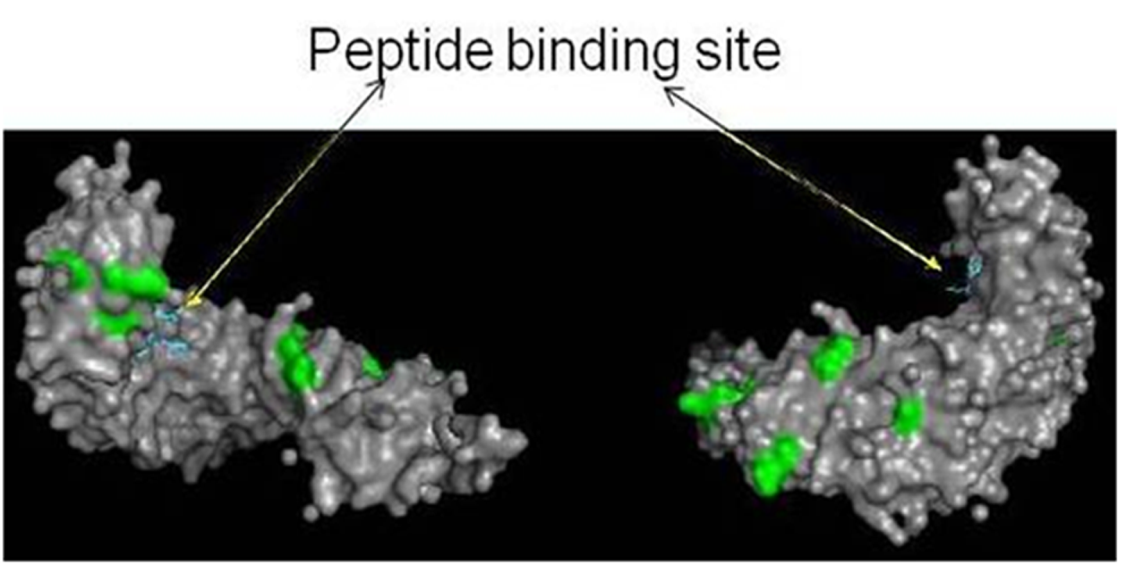


Figure S4. Mapping of positively selected amino-acids onto the HcpC crystal structure. The peptide binding groove in the HcpC molecular surface is indicated.


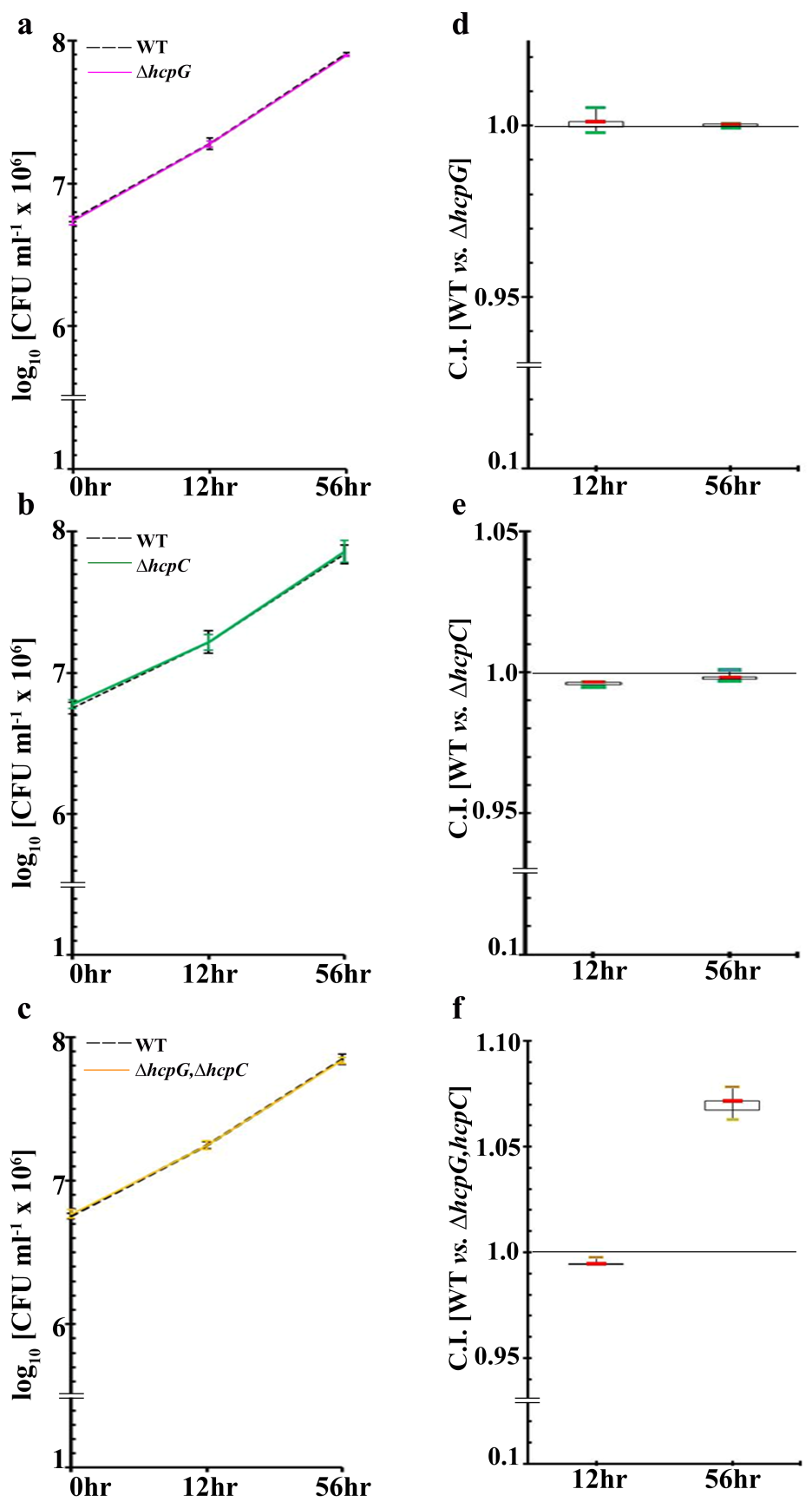


Figure S5. Deletion of *hcpG* and/or *hcpG* does not affect *H. pylori* fitness in broth cultures. (a, b, and c) CFUs per milliliter of each *hcp* mutant co-cultured with the WT strain of G27MA. (c, e, and f) The corresponding CIs for each mutant-WT co-culture experiment. Results show average of three experiments.

**
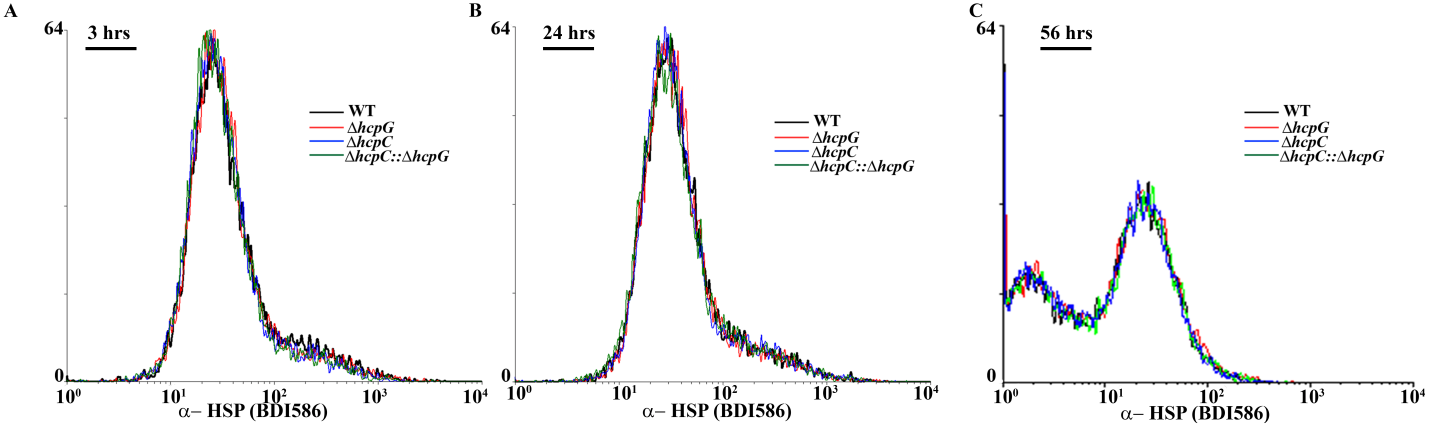
**

Figure S6. Deletion of *hcpG* and/or *hcpC* does not affect HspB expression in broth culture. Histograms showing comparable anti-HspB fluorescence in WT and mutant strains in pure BHI-broth cultures at 3, 24, and 56 h of growth. Note: in Panel C, anti-HspB antibody was used at a concentration of 0.5 μg/mL. Results are representative of three experiments.

Supplementary Tables

| **Table S1. *H. pylori* isolates and their geographic origins.** | | |
| --- | --- | --- |
| **Strain** | **Origin** | **Disease** |
| GAM4655 | The Gambia | n.d. |
| GAM4842 | The Gambia | n.d. |
| GAM4636 | The Gambia | n.d. |
| GAM9419 | The Gambia | n.d. |
| JF28 | Fukui, Japan | n.d. |
| JF55 | Fukui, Japan | n.d. |
| JF58 | Fukui, Japan | n.d. |
| JF79 | Fukui, Japan | n.d. |
| JS03 | Japan | n.d. |
| JS07 | Japan | n.d. |
| JS08 | Japan | n.d. |
| JS10 | Japan | n.d. |
| JS11 | Japan | n.d. |
| JS12 | Japan | n.d. |
| CPY2362 | Japan | Gastritis |
| CPY6021 | Japan | Gastritis |
| HU29 | Honshu, Japan | Gastritis |
| HU38 | Honshu, Japan | Gastritis |
| HU56 | Honshu, Japan | Gastritis |
| HU78 | Honshu, Japan | Gastritis |
| HU87 | Honshu, Japan | Gastritis |
| HU131 | Honshu, Japan | Gastritis |
| HU133 | Honshu, Japan | Gastritis |
| HU157 | Honshu, Japan | Gastric Cancer |
| CPY6271 | Japan | Gastric Cancer |
| CPY6311 | Japan | Gastric Cancer |
| CPY6261 | Japan | Gastric Cancer |
| HU176 | Honshu, Japan | Gastric Cancer |
| HU178 | Honshu, Japan | Gastric Cancer |
| HU54 | Honshu, Japan | Gastric Cancer |
| HU118 | Honshu, Japan | Gastric Cancer |
| HU48 | Honshu, Japan | Gastric Cancer |
| HU71 | Honshu, Japan | Gastric Cancer |
| CPY6081 | Japan | Gastric Cancer |
| K08 | South Korea | n.d. |
| K17 | South Korea | n.d. |
| K24 | South Korea | n.d. |
| K25 | South Korea | n.d. |
| K30 | South Korea | n.d. |
| K42 | South Korea | n.d. |
| K51 | South Korea | n.d. |
| K65 | South Korea | n.d. |
| K120 | South Korea | n.d. |
| R07 | South Africa | n.d. |
| R10 | South Africa | n.d. |
| R40 | South Africa | n.d. |
| R50 | South Africa | n.d. |
| S24 | Spain | n.d. |
| S45 | Spain | n.d. |
| S46 | Spain | n.d. |
| S47 | Spain | n.d. |
| S57 | Spain | n.d. |
| HUP-B43 | Spain | n.d. |
| HUP-B45 | Spain | n.d. |
| HUP-B46 | Spain | n.d. |
| HUP-B53 | Spain | n.d. |
| HUP-B56 | Spain | n.d. |
| HUP-B60 | Spain | n.d. |
| HUP-B65 | Spain | n.d. |
| HUP-B70 | Spain | n.d. |
| HUP-B76 | Spain | n.d. |
| HUP-B80 | Spain | n.d. |
| HUP-B82 | Spain | n.d. |
| HUP-B84 | Spain | n.d. |
| HUP-B41 | Spain | n.d. |
| HUP-B49 | Spain | n.d. |
| HUP-B50 | Spain | n.d. |
| HUP-B51 | Spain | n.d. |
| HUP-B58 | Spain | n.d. |
| HUP-B59 | Spain | n.d. |
| HUP-B62 | Spain | n.d. |
| HUP-B63 | Spain | n.d. |
| HUP-B71 | Spain | n.d. |
| HUP-B79 | Spain | n.d. |
| HUP-B85 | Spain | n.d. |
| HUP-B86 | Spain | n.d. |
| SJM9 | Lima, Peru | Gastritis |
| SJM26 s.c. | Lima, Peru | Gastritis |
| SJM42 s.c. | Lima, Peru | Gastritis |
| SJM68 s.c. | Lima, Peru | Gastritis |
| SJM75 s.c. | Lima, Peru | Gastritis |
| SJM79 s.c. | Lima, Peru | Gastritis |
| SJM91 s.c. | Lima, Peru | Gastritis |
| SJM148 s.c. | Lima, Peru | Gastritis |
| SJM179 b.c. | Lima, Peru | Gastritis |
| SJM180A | Lima, Peru | Gastritis |
| SJM184A | Lima, Peru | Gastritis |
| SJM189A | Lima, Peru | Gastritis |
| PeCan1B | Lima, Peru | Gastric Cancer |
| PeCan2A | Lima, Peru | Gastric Cancer |
| PeCan4A | Lima, Peru | Gastric Cancer |
| PeCan9A | Lima, Peru | Gastric Cancer |
| PeCan10B | Lima, Peru | Gastric Cancer |
| PeCan14B | Lima, Peru | Gastric Cancer |
| PeCan16A | Lima, Peru | Gastric Cancer |
| PeCan18B | Lima, Peru | Gastric Cancer |
| PeCan22A | Lima, Peru | Gastric Cancer |
| PeCan28B | Lima, Peru | Gastric Cancer |
| PeCan32A | Lima, Peru | Gastric Cancer |
| PeCan38B | Lima, Peru | Gastric Cancer |
| I-7A | Kolkata, India | n.d. |
| I-27B s.c. | . | n.d. |
| I-34B s.c. | Kolkata, India | n.d. |
| I-39A b.c. | Kolkata, India | n.d. |
| I-44A | Kolkata, India | n.d. |
| I-48B | Kolkata, India | n.d. |
| I-49 | Kolkata, India | n.d. |
| I-56B b.c. | Kolkata, India | n.d. |
| I-66B s.c. | Kolkata, India | n.d. |
| I-67B | Kolkata, India | n.d. |
| I-75A s.c. | Kolkata, India | n.d. |
| I-77A | Kolkata, India | n.d. |
| Santal10 | Kolkata, India | n.d. |
| Santal31 | Kolkata, India | n.d. |
| Santal49 | Kolkata, India | n.d. |
| Santal51 | Kolkata, India | n.d. |
| Santal52 | Kolkata, India | n.d. |
| Santal54 | Kolkata, India | n.d. |
| Chen1 | Chennai, India | n.d. |
| Chen2 | Chennai, India | n.d. |
| Chen3 | Chennai, India | n.d. |
| Chen4 | Chennai, India | n.d. |
| 12Acol2 | Shimaa (Ethnic Peruvian) | n.d. |
| 17Acol2 | Shimaa (Ethnic Peruvian) | n.d. |
| 18Acol2 | Shimaa (Ethnic Peruvian) | n.d. |
| 31Acol2 | Shimaa (Ethnic Peruvian) | n.d. |
| 32Acol2 | Shimaa (Ethnic Peruvian) | n.d. |
| 33Acol2 | Shimaa (Ethnic Peruvian) | n.d. |
| 35Acol2 | Shimaa (Ethnic Peruvian) | n.d. |
| 36Acol2 | Shimaa (Ethnic Peruvian) | n.d. |
| 46Acol2 | Shimaa (Ethnic Peruvian) | n.d. |
| 49Acol2 | Shimaa (Ethnic Peruvian) | n.d. |
| 62Acol2 | Shimaa (Ethnic Peruvian) | n.d. |
| 71Acol2 | Shimaa (Ethnic Peruvian) | n.d. |
| 73Acol2 | Shimaa (Ethnic Peruvian) | n.d. |
| 74Acol2 | Shimaa (Ethnic Peruvian) | n.d. |
| 112Acol2 | Shimaa (Ethnic Peruvian) | n.d. |
| 117Acol2 | Shimaa (Ethnic Peruvian) | n.d. |
| 156Acol2 | Shimaa (Ethnic Peruvian) | n.d. |
| 169Acol2 | Shimaa (Ethnic Peruvian) | n.d. |
| 170Acol2 | Shimaa (Ethnic Peruvian) | n.d. |
| 193Acol2 | Shimaa (Ethnic Peruvian) | n.d. |
| 216Acol2 | Shimaa (Ethnic Peruvian) | n.d. |
| 222Acol2 | Shimaa (Ethnic Peruvian) | n.d. |
| 236Acol2 | Shimaa (Ethnic Peruvian) | n.d. |
| 243Acol2 | Shimaa (Ethnic Peruvian) | n.d. |
| 249Acol2 | Shimaa (Ethnic Peruvian) | n.d. |
| 257Acol2 | Shimaa (Ethnic Peruvian) | n.d. |
| 258Acol1 | Shimaa (Ethnic Peruvian) | n.d. |
| 304Acol2 | Shimaa (Ethnic Peruvian) | n.d. |
| 329Acol2 | Shimaa (Ethnic Peruvian) | n.d. |
| 399Acol2 | Shimaa (Ethnic Peruvian) | n.d. |
| 416Acol2 | Shimaa (Ethnic Peruvian) | n.d. |
| 417Acol2 | Shimaa (Ethnic Peruvian) | n.d. |
| 439Acol2 | Shimaa (Ethnic Peruvian) | n.d. |
| 463Acol2 | Shimaa (Ethnic Peruvian) | n.d. |
| 1col1 | Shimaa (Ethnic Peruvian) | n.d. |
| 30Acol1 | Shimaa (Ethnic Peruvian) | n.d. |
| 126Acol1 | Shimaa (Ethnic Peruvian) | n.d. |
| 217Acol1 | Shimaa (Ethnic Peruvian) | n.d. |
| 248Acol1 | Shimaa (Ethnic Peruvian) | n.d. |
| 309col1 | Shimaa (Ethnic Peruvian) | n.d. |
| 314col1 | Shimaa (Ethnic Peruvian) | n.d. |
| 384Acol1 | Shimaa (Ethnic Peruvian) | n.d. |
| 429Acol1 | Shimaa (Ethnic Peruvian) | n.d. |
| 470Acol1 | Shimaa (Ethnic Peruvian) | n.d. |

| **Table S2. Nucleotide sequences of PCR primers used in this study.** | | | | | | |
| --- | --- | --- | --- | --- | --- | --- |
| **Primer name** | **nt sequence (5' → 3')** | | **Primer location [Gene: Target nt]** | | **Product Size** | **Application** |
| ***hcpC* PCR amplification and nt sequencing** | | | | | | |
| hcpCF1 | | CGCCTTCGCCATTGTATTGCATA | | *Jhp1023*: 747-769 | 1643 bp | PCR amplification and sequencing |
| hcpCR1 | | GCCCCGGGTCTTACGATAAAGCTTTTAGAACA | | *Jhp1025*: 269-299 |  |  |
| 1098F | | GGGTGTTTTAATTTAGGGGTGCT | | *jhp1024*: 200-223 |  | internal sequencing |
| ***hcpG* PCR amplification and nt sequencing** | | | | | | |
| hcpGF1 | | CATTTGTTGGATATTGTGGG | | *jhp1436*: 274-293 | 988 bp | PCR amplification and sequencing |
| hcpGR1 | | AGGACAAAGGGTTTGTT | | *jhp1438*: 26-42 |  |  |
| hcpGF2 | | TCAAAAAAGCGGTTTTTAGGG | | *jhp1437*: 11-31 | 750 bp | Internal fragment amplification, sequencing |
| hcpGR2 | | TTGTTCTTTGAGAATATCGC | | *jhp1437*:743-762 |  |  |
| ***hcpC assembly PCRs for generating ΔhcpC strains*** | | | | | | |
| 1098A1n [1]* | | CGCCTTCGCCATTGTATTGCATA | | *hp1098*: 747-769 | 509 bp | Fragment A amplification |
| 1098A2n [2]* | | *gtgtttaatccatagttataaagcat*CCAGCAATGGGTGTCAATTTGCTAGGGA | | *hp1098*:288-313 |  |  |
| 1098C5n [3]* | | *ttcaatagctataaattatttaataagtaa*CAAGCACAAAACCCTAAAAAAGGACT | | *hp1098*: 20-47 | 500 bp | Fragment C amplification |
| 1098C6n [4]* | | GCCCCGGGTCTTACGATAAAGCTTTTAGAACA | | *hp1099*: 269-299 |  |  |
| rpsL-F [5]* | | GATGCTTTATAACTATGGATTAAACAC^¶^ | | *rpsL,erm*: 1-27 | 1539 bp | Fragment B, *rpsL,erm* casette, amplfication |
| erm-R [6]* | | TTACTTATTAAATAATTTATAGCTATTGAA^¶^ | | *rpsL,erm*: 1510-1539 |  |  |
| ***hcpG assembly PCRs for generating ΔhcpG strains & hcpG::6XHis fusion construct*** | | | | | | |
| hcpGAF [1]* | | GAAGTGCTGTATTATAAGGATT | | *jhp1435*: 987-1008 | 700 bp | Fragment A amplification |
| hcpGAR2 [2]* | | *gatcgaattc*CCGACACTCTCCTCCCTTTTGTAA | | 1584828-1584852^‡^ |  |  |
| hcpGCF2 [3]* | | *gatcgcggccgc*AAATCGTAGCCCTATTAAGCCCCAT | | 1585624-1585648^‡^ | 750 bp | Fragment C amplification |
| hcpGCR [4]* | | TATTTAAAAGCGTGCGGGCTTA | | *jhp1438*: 716-737 |  |  |
| rpsLF2 [5]* | | *gatcgaattc*GGATGCTTTATAACTATAACTATGATT**^∫^** | | *rpsL,cat*: 1-26 | 1500 bp | Fragment B, *rpsL,cat* casette, amplification |
| **Primer name** | | **nt sequence (5' → 3')** | | **Primer location [Gene: Target nt]** | **Product Size** | **Application** |
| catR2 [6]* | | *gatcgcggccgc*TTATCAGTGCGACAAACTGGG**^∫^** | | *rpsL,erm*: 1395-1420 |  |  |
| hcpGBF [5']† | | *gatcgaattc*ATGTTAAGGGGTGTCAAAAAAGC | | *hpG27_1469*: 1-23 | 984 bp | *hcpG*:*:6Xhis* amplification |
| hcpGBR [6']† | | *gatcgcggccgcatgatggtggtgatgat*GTTATTGTTTTTGTTCTTTGAG | | *hpG27_1469*: 960-984 |  |  |
| ***Reverse transcription PCRs*** | | | | | | |
| hcpCF-cDNA | TGGCAGAGCAAGACCCTAAAGA | | | *hp1098*: 782-803 | 245 bp | *hcpC*-transcript amplification |
| hcpCR-cDNA | ATTCCCTAGCAAATACACC | | | *hp1098*: 559-578 |  |  |
|  |  | | |  |  |  |
| hcpGF-cDNA | AGAAGGCTGGGGCTCATTTG | | | *jhp1437*: 152-172 | 247 bp | *hcpG* transcript amplification |
| hcpGR-cDNA | TTACAAGATTTAGAGTAATATT | | | *jhp1437*: 377-398 |  |  |
| ureAF | ATGAAACTCACCCCAAAAGAGTT | | | *ureA*: 65-771 | 271 bp | *ureA* transcript amplification |
| ureAR | CACATCATCCGGTTTTAAAAGA | | | *ureA*: 501-529 |  |  |
| recAF | GCAATAGATGAAGACAAACA | | | *recA*: 4-23 | 206 bp | *recA* transcript amplification [control] |
| recAR | GACTCTGGCCCATAAATTTCA | | | *recA*: 180-209 |  |  |
| GAPDHF | AGAAGGCTGGGGCTCATTTG | | | *gapdh*: 369-389 | 278 bp | *gapdh* transcript amplification [control] |
| GAPDHR | AGGGGCCATCCACAGTCTTC | | | *gapdh*: 626-646 |  |  |
| ***Quantitative Real-time PCRs*** | | | | | | |
| jhp1024F | GTGCCTCTGCCTGCATCAT | | | *jhp1024*: 378-396 | 109 bp | *hcpC*-transcript |
| jhp1024R | TTTGAACGATGGCGATGGT | | | *jhp1024*: 468-487 |  |  |
| jhp1437F | AGGCGTGGCAAAGGATGA | | | *jhp1437*: 152-172 | 109 bp | *hcpG*-transcript |
| jhp1437R | AATCCTAGCTTGCAACTCTTTTCAA | | | *jhp1437*: 756-777 |  |  |
| groelF | TGTGGTTAAAAGCAGGCTTGAA | | | *groEL:* 99-121 | 102 bp | *hspB*-transcript |
| groelR | GGCATTATTGACCCCTTAAAAGTAGA | | | *groEL*: 179-201 |  |  |
| ureBF | GTTCGCATCACCCATTTGACT | | | *ureB*: 318-339 | 110 bp | *ureB*-transcript |
| urebR | GCGTGAAACCTAACATGATCATCA | | | *ureB*: 408-428 |  |  |
| **Primer name** | **nt sequence (5' → 3')** | | | **Primer location [Gene: Target nt]** | **Product Size** | **Application** |
| recAF1 | TGCAGAATGCCAAAAAAATGG | | | *recA*: 924-944 | 100 bp | *recA*-transcript [control] |
| recAR1 | AAGGCATGCTCAGCGTCAA | | | *recA*: 1004-1024 |  |  |
| ureAF1 | CCATCCATCACATCATCTGGTT | | | *ureA*: 502-523 | 104 bp | *ureA*-transcript [control] |
| ureAR1 | TGCGGCTGAATTGATGCA | | | *ureA*: 587-606 |  |  |
| *, Primer numbers in square brackets correspond to primer designations shown in supplementary figure SM1 | | | | | |  |
| ¶, Described in Dailidiene et al 2006 | | | |  |  |  |
| ∫, Described in Shen et al 2010 | | | |  |  |  |
| ‡, HpJ99 genomic co-ordinates, | | | |  |  |  |
| †, Alternate primers used for construction of *hcpG::6Xhis* assembly or fragment B' | | | | |  |  |
| Restriction sites in primers are indicated by single underlines; double underlines indicate sequence corresponding to the 6xHis tag. | | | | | | |

| **Table S3. Model Selection and Initial Model Parameters for *hcpG* Phylogenetic Reconstruction.** | | | | | | | | | | | | |  |  |  |  |  |  |
| --- | --- | --- | --- | --- | --- | --- | --- | --- | --- | --- | --- | --- | --- | --- | --- | --- | --- | --- |
| **Null Model** | **InL0** | | **Alternative Model** | | **InL1** | | **hLRT*** | | **DF** | | **P-Value**** | |  |  | | |  |  |
|  |  | |  | |  | |  | |  | |  | |  |  | | |  |  |
| **Test: Equal Base Frequencies.** | | | | |  | |  | |  | |  | |  |  | | |  |  |
| JC | -5277.99 | | F81 | | -5192.959 | | 170.0664 | | 3 | | <0.000001 | | |  | | |  |  |
|  |  | |  | |  | |  | |  | |  | |  |  | | |  |  |
| **Test: Transition Rate = Transversion Rate.** | | | | | | |  | |  | |  | |  |  | | |  |  |
| F81 | 5192.959 | | HKY85 | | -4921.189 | | 543.54 | | 1 | | <0.000001 | | |  | | |  |  |
|  |  | |  | |  | |  | |  | |  | |  |  | | |  |  |
| **Test: Equal Transition Rates.** | | | | |  | |  | |  | |  | |  |  | | |  |  |
| HKY85 | 4921.189 | | TrN | | 4918.4307 | | 5.5166 | | 1 | | <0.05 | |  |  | | |  |  |
|  |  | |  | |  | |  | |  | |  | |  |  | | |  |  |
| **Test: Equal Transversion Rates.** | | | | |  | |  | |  | |  | |  |  | | |  |  |
| HKY85 | 4921.189 | | K81uf | | 4913.7017 | | 14.9746 | | 1 | | <0.0001 | |  |  | | |  |  |
|  |  | |  | |  | |  | |  | |  | |  |  | | |  |  |
| **Test: Only two Transversion Rates.** | | | | | | |  | |  | |  | |  |  | | |  |  |
| K81uf | 4913.702 | | TVM | | 4907.8599 | | 11.6836 | | 2 | | <0.001 | |  |  | | |  |  |
|  |  | |  | |  | |  | |  | |  | |  |  | | |  |  |
| **Test: Equal Rates Among Sites.** | | | | |  | |  | |  | |  | |  |  | | |  |  |
| TVM | 4907.86 | | TVM+G | | 4716.9077 | | 381.9043 | | 1 | | <0.000001 | | |  | | |  |  |
|  |  | |  | |  | |  | |  | |  | |  |  | | |  |  |
| **Test: No Invariable Sites.** | | | | |  | |  | |  | |  | |  |  | | |  |  |
| TVM+G | 4716.908 | | TVM+I+G | | 4716.9077 | | 0 | | 1 | | >0.999999 | |  |  | | |  |  |
| * Hierarchial Likelihood Ratio Test | | | | | | |  | |  | |  | |  |  | | |  |  |
| ** Confidence-Level: 0.01 | | | | |  | |  | |  | |  | |  |  | | |  |  |
| InL, Log-Likelihood | | |  | |  | |  | |  | |  | |  |  | | |  |  |
| DF, Degrees of Freedom | | | | |  | |  | |  | |  | |  |  | | |  |  |
| **Model Selected: TVM+G** | | | | | **Substitution model:** | | | | **Rate matrix** | | | |  |  | | |  |  |
| -lnL = | 4716.908 | |  | | R(a) [A-C] = | |  | |  | | 1.7069 | |  |  | | |  |  |
| K = | 8 | |  | | R(b) [A-G] = | |  | |  | | 7.3749 | |  |  | | |  |  |
| Base frequencies: | | |  | | R(c) [A-T] = | |  | |  | | 0.4896 | |  |  | | |  |  |
| freqA = | 0.36 | |  | | R(d) [C-G] = | |  | |  | | 0.99 | |  |  | | |  |  |
| freqC = | 0.1335 | |  | | R(e) [C-T] = | |  | |  | | 7.3749 | |  |  | | |  |  |
| freqG = | 0.2217 | |  | | R(f) [G-T] = | |  | |  | | 1 | |  |  | | |  |  |
| freqT = | 0.2848 | |  | |  | |  | |  | |  | |  |  | | |  |  |
| **Among-site rate variation** | | | | | **Variable sites (G)** | | | |  | |  | |  |  | | |  |  |
| I= | 0 | |  | | α= | | 0.4023 | |  | |  | |  |  | | |  |  |
| **Table S4. Model Selection and Initial Model Parameters for *hcpC* Phylogenetic Reconstruction.** | | | | | | | | | | | | | | |  |  |  |  |
| **Null Model** | | **InL0** | | **Alternative Model** | | **InL1** | | **hLRT*** | | **DF** | | **P-Value**** | | | |  | |  |
|  | |  | |  | |  | |  | |  | |  | | | |  | |  |
| **Test: Equal Base Frequencies.** | | | | | |  | |  | |  | |  | | | |  | |  |
| JC | | -7516.066 | | F81 | | 7480.761 | | 70.6094 | | 3 | | <0.000001 | | | | | |  |
|  | |  | |  | |  | |  | |  | |  | | | |  | |  |
| **Test: Transition Rate = Transversion Rate.** | | | | | | | |  | |  | |  | | | |  | |  |
| F81 | | 7480.762 | | HKY85 | | 7057.8271 | | 845.869 | | 1 | | <0.000001 | | | | | |  |
|  | |  | |  | |  | |  | |  | |  | | | |  | |  |
| **Test: Equal Transition Rates.** | | | | | |  | |  | |  | |  | | | |  | |  |
| HKY85 | | 7057.827 | | TrN | | 7004.728 | | 106.1982 | | 1 | | <0.000001 | | | | | |  |
|  | |  | |  | |  | |  | |  | |  | | | |  | |  |
| **Test: Equal Transversion Rates.** | | | | | |  | |  | |  | |  | | | |  | |  |
| TrN | | 7004.728 | | TIM | | 7001.8823 | | 5.691 | | 1 | | <0.05 | | | |  | |  |
|  | |  | |  | |  | |  | |  | |  | | | |  | |  |
| **Test: Equal Rates Among Sites.** | | | | | |  | |  | |  | |  | | | |  | |  |
| TrN | | 7004.728 | | Trn+Γ | | 5992.6396 | | 2024.177 | | 1 | | <0.000001 | | | | | |  |
|  | |  | |  | |  | |  | |  | |  | | | |  | |  |
| **Test: No Invariable Sites.** | | | | | |  | |  | |  | |  | | | |  | |  |
| TrN+Γ | | 5992.64 | | TrN+I+Γ | | 5981.2578 | | 22.7637 | | 1 | | <0.000001 | | | |  | |  |
|  | |  | |  | |  | |  | |  | |  | | | |  | |  |
| **See footnotes for Table S2.** | | | | | |  | |  | |  | |  | | | |  | |  |
| **Model Selected: TrN+I+Γ** | | | | | | **Substitution model:** | | | | **Rate matrix** | | | | | |  | |  |
| -lnL = | | 5981.258 | |  | | R(a) [A-C] = | |  | |  | | 1 | | | |  | |  |
| K = | | 7 | |  | | R(b) [A-G] = | |  | |  | | 6.5621 | | | |  | |  |
| Base frequencies: | | | |  | | R(c) [A-T] = | |  | |  | | 1 | | | |  | |  |
| freqA = | | 0.3143 | |  | | R(d) [C-G] = | |  | |  | | 1 | | | |  | |  |
| freqC = | | 0.1567 | |  | | R(e) [C-T] = | |  | |  | | 11.8894 | | | |  | |  |
| freqG = | | 0.2712 | |  | | R(f) [G-T] = | |  | |  | | 1 | | | |  | |  |
| freqT = | | 0.2578 | |  | |  | |  | |  | |  | | | |  | |  |
| **Among-site rate variation** | | | | | | **Variable sites (Γ)** | | | |  | |  | | | |  | |  |
| I= | | 0.5367 | |  | | α | | 0.3544 | |  | |  | | | |  | |  |

| **Table S5. FACS Parameters used in this study.** | | |
| --- | --- | --- |
|  | **α-HSP** | **α-CagA/pSer MAPK** |
| Forward Scatter (FSC) | E00 | E00 |
| Side Scatter (SSC) | 387 | 408 |
| Florescence Channel 1 (FL1) | 412 | 364 |
| Florescence Channel 2 (FL2) | 150 | 400 |
| Florescence Channel 3 (FL3) | 150 | 150 |
| Florescence Channel 4 (FL4) | 150 | 819 |

| **Table S6. Occurrence of *hcpG* and its similarity with *hcpC* in fully sequenced *H. pylori* genomes** | | | | | | | | |
| --- | --- | --- | --- | --- | --- | --- | --- | --- |
| **Strain** | ***hcpC* annotation** | ***hcpG* annotation** | **nt Identity / Similarity[%]** | **Query coverage**  **[%]** | **Expect-Value** | **AA Identity / Similarity [%]** | **Query coverage**  **[%]** | **Expect-Value** |
| JHp99 | jhp1024 | jhp1437 | NSS^¶^ | NA | NA | 41/53 | 98 | 7.00E-52 |
| Hp26695 | hp1098 | *Absent* | - | - | - | - | - | - |
| HpG27 | HpG27_1039 | HpG27_1469***** | 90 | 9 | 5.00E-29 | 44/58 | 98 | 2.00E-71 |
| HPAG1 | hpag1_1036 | *Absent* | - | - | - | - | - | - |
| SHI470 | HpSH_05640 | *Absent* | - | - | - | - | - | - |
| P12 | HPP12_1063 | *Absent* | - | - | - | - | - | - |
| B8 | HpB8_406 | HpB8_1690 | NSS | NA | NA | 44/59 | 98 | 1.00E-71 |
| Hp51 | KHp_0998 | *Absent* | - | - | - | - | - | - |
| Hp52 | KHpB_1028 | *Absent* | - | - | - | - | - | - |
| HpV225d | HPV225_1116 | HPV225_1471 | NSS | NA | NA | 43/60 | 100 | 2.00E-34 |
| ¶, NSS - No Significant Similarity found by BLASTN | | | |  |  |  |  |  |
| ***, In strain HPG27, HpG27_1469 is misannotated as HcpC** | | | | | | | | |

| **Table S7: dN/dS for each *hcpG* lineage computed by the M1bra model.** | | | | | | |  |  |
| --- | --- | --- | --- | --- | --- | --- | --- | --- |
| **Branch** | **t** | **N** | **S** | **dN/dS** | **dN** | **dS** | **N*dN** | **S*dS** |
| 47..1 | 0.003 | 722.9 | 261.1 | 144.9719 | 0.0014 | 0 | 1 | 0 |
| 47..48 | 0.02 | 722.9 | 261.1 | 0.1671 | 0.0028 | 0.0169 | 2 | 4.4 |
| 48..49 | 0.006 | 722.9 | 261.1 | 0.0001 | 0 | 0.0077 | 0 | 2 |
| 49..50 | 0.007 | 722.9 | 261.1 | 0.0001 | 0 | 0.009 | 0 | 2.3 |
| 50..51 | 0 | 722.9 | 261.1 | 3.5519 | 0 | 0 | 0 | 0 |
| 51..52 | 0.003 | 722.9 | 261.1 | 101.6999 | 0.0014 | 0 | 1 | 0 |
| 52..53 | 0.028 | 722.9 | 261.1 | 0.7037 | 0.0084 | 0.012 | 6.1 | 3.1 |
| 53..54 | 0.003 | 722.9 | 261.1 | 0.0001 | 0 | 0.0037 | 0 | 1 |
| 54..55 | 0.005 | 722.9 | 261.1 | 0.8995 | 0.0015 | 0.0017 | 1.1 | 0.4 |
| 55..56 | 0.031 | 722.9 | 261.1 | 0.3227 | 0.0066 | 0.0204 | 4.8 | 5.3 |
| 56..57 | 0.058 | 722.9 | 261.1 | 0.4633 | 0.0148 | 0.032 | 10.7 | 8.4 |
| 57..58 | 0.017 | 722.9 | 261.1 | 0.6004 | 0.0048 | 0.008 | 3.5 | 2.1 |
| 58..59 | 0.297 | 722.9 | 261.1 | 0.3259 | 0.064 | 0.1964 | 46.3 | 51.3 |
| 59..60 | 0.028 | 722.9 | 261.1 | 147.9977 | 0.0126 | 0.0001 | 9.1 | 0 |
| 60..61 | 0.015 | 722.9 | 261.1 | 120.9004 | 0.0066 | 0.0001 | 4.8 | 0 |
| 61..62 | 0.005 | 722.9 | 261.1 | 99.9328 | 0.0023 | 0 | 1.6 | 0 |
| 62..2 | 0.134 | 722.9 | 261.1 | 0.8989 | 0.0435 | 0.0484 | 31.5 | 12.6 |
| 62..63 | 0.005 | 722.9 | 261.1 | 0.0001 | 0 | 0.0067 | 0 | 1.7 |
| 63..64 | 0.043 | 722.9 | 261.1 | 0.9909 | 0.0141 | 0.0143 | 10.2 | 3.7 |
| 64..9 | 0 | 722.9 | 261.1 | 0.0001 | 0 | 0 | 0 | 0 |
| 64..29 | 0 | 722.9 | 261.1 | 0.0001 | 0 | 0 | 0 | 0 |
| 63..27 | 0.064 | 722.9 | 261.1 | 0.6809 | 0.019 | 0.0279 | 13.7 | 7.3 |
| 61..28 | 0.021 | 722.9 | 261.1 | 1.0488 | 0.0072 | 0.0069 | 5.2 | 1.8 |
| 60..30 | 0.044 | 722.9 | 261.1 | 0.3383 | 0.0096 | 0.0284 | 6.9 | 7.4 |
| 59..38 | 0.047 | 722.9 | 261.1 | 0.0637 | 0.0032 | 0.0506 | 2.3 | 13.2 |
| 58..65 | 0.039 | 722.9 | 261.1 | 0.6699 | 0.0114 | 0.0171 | 8.3 | 4.5 |
| 65..7 | 0.148 | 722.9 | 261.1 | 0.4398 | 0.0368 | 0.0836 | 26.6 | 21.8 |
| 65..25 | 0.095 | 722.9 | 261.1 | 0.3063 | 0.0197 | 0.0645 | 14.3 | 16.8 |
| 57..66 | 0.037 | 722.9 | 261.1 | 0.1933 | 0.0058 | 0.0299 | 4.2 | 7.8 |
| 66..13 | 0.034 | 722.9 | 261.1 | 0.4279 | 0.0083 | 0.0194 | 6 | 5.1 |
| 66..20 | 0 | 722.9 | 261.1 | 5.0999 | 0 | 0 | 0 | 0 |
| 56..3 | 0.013 | 722.9 | 261.1 | 0.1562 | 0.0017 | 0.011 | 1.2 | 2.9 |
| 55..67 | 0.008 | 722.9 | 261.1 | 87.9038 | 0.0036 | 0 | 2.6 | 0 |
| 67..6 | 0.081 | 722.9 | 261.1 | 0.4812 | 0.0211 | 0.0438 | 15.3 | 11.4 |
| 67..68 | 0.087 | 722.9 | 261.1 | 0.3603 | 0.0198 | 0.0549 | 14.3 | 14.3 |
| 68..10 | 0.264 | 722.9 | 261.1 | 1.3102 | 0.0939 | 0.0716 | 67.9 | 18.7 |
| 68..69 | 0.151 | 722.9 | 261.1 | 0.666 | 0.0444 | 0.0666 | 32.1 | 17.4 |
| 69..23 | 0.284 | 722.9 | 261.1 | 0.7219 | 0.086 | 0.1192 | 62.2 | 31.1 |
| 69..37 | 0.043 | 722.9 | 261.1 | 0.1399 | 0.0055 | 0.0392 | 4 | 10.2 |
| 67..46 | 0.031 | 722.9 | 261.1 | 0.1058 | 0.0032 | 0.0301 | 2.3 | 7.9 |
| 54..70 | 0.171 | 722.9 | 261.1 | 0.1554 | 0.0233 | 0.1501 | 16.9 | 39.2 |
| 70..4 | 0 | 722.9 | 261.1 | 2.1344 | 0 | 0 | 0 | 0 |
| 70..26 | 0.01 | 722.9 | 261.1 | 148.2086 | 0.0043 | 0 | 3.1 | 0 |
| 53..71 | 0.006 | 722.9 | 261.1 | 75.6537 | 0.0028 | 0 | 2 | 0 |
| **Branch** | **t** | **N** | **S** | **dN/dS** | **dN** | **dS** | **N*dN** | **S*dS** |
| 71..12 | 0.016 | 722.9 | 261.1 | 0.2359 | 0.0028 | 0.0119 | 2 | 3.1 |
| 71..72 | 0.013 | 722.9 | 261.1 | 0.3162 | 0.0027 | 0.0086 | 2 | 2.3 |
| 72..16 | 0.047 | 722.9 | 261.1 | 0.1613 | 0.0066 | 0.0406 | 4.7 | 10.6 |
| 72..39 | 0.041 | 722.9 | 261.1 | 0.327 | 0.0087 | 0.0267 | 6.3 | 7 |
| 52..73 | 0.009 | 722.9 | 261.1 | 147.5343 | 0.0042 | 0 | 3 | 0 |
| 73..74 | 0.003 | 722.9 | 261.1 | 0.0001 | 0 | 0.0039 | 0 | 1 |
| 74..75 | 0.009 | 722.9 | 261.1 | 0.8738 | 0.0028 | 0.0032 | 2 | 0.8 |
| 75..21 | 0.036 | 722.9 | 261.1 | 0.1257 | 0.0042 | 0.0333 | 3 | 8.7 |
| 75..24 | 0.01 | 722.9 | 261.1 | 0.1637 | 0.0014 | 0.0085 | 1 | 2.2 |
| 74..42 | 0.009 | 722.9 | 261.1 | 0.1776 | 0.0014 | 0.0079 | 1 | 2.1 |
| 74..43 | 0.012 | 722.9 | 261.1 | 0.119 | 0.0014 | 0.0118 | 1 | 3.1 |
| 73..22 | 0.019 | 722.9 | 261.1 | 0.0001 | 0 | 0.0235 | 0 | 6.1 |
| 73..76 | 0.006 | 722.9 | 261.1 | 0.0001 | 0 | 0.0078 | 0 | 2 |
| 76..35 | 0.006 | 722.9 | 261.1 | 0.3621 | 0.0014 | 0.0039 | 1 | 1 |
| 76..77 | 0.01 | 722.9 | 261.1 | 0.1462 | 0.0013 | 0.0086 | 0.9 | 2.2 |
| 77..40 | 0.052 | 722.9 | 261.1 | 0.1171 | 0.0058 | 0.0497 | 4.2 | 13 |
| 77..45 | 0.019 | 722.9 | 261.1 | 2.3096 | 0.0073 | 0.0032 | 5.3 | 0.8 |
| 52..78 | 0.009 | 722.9 | 261.1 | 0.7102 | 0.0028 | 0.0039 | 2 | 1 |
| 78..34 | 0.019 | 722.9 | 261.1 | 0.0001 | 0 | 0.0236 | 0 | 6.2 |
| 78..36 | 0.028 | 722.9 | 261.1 | 0.4428 | 0.007 | 0.0157 | 5 | 4.1 |
| 51..79 | 0.006 | 722.9 | 261.1 | 0.3617 | 0.0014 | 0.0039 | 1 | 1 |
| 79..80 | 0.006 | 722.9 | 261.1 | 0.3542 | 0.0014 | 0.004 | 1 | 1 |
| 80..5 | 0.015 | 722.9 | 261.1 | 0.5544 | 0.0042 | 0.0077 | 3.1 | 2 |
| 80..41 | 0.009 | 722.9 | 261.1 | 0.0001 | 0 | 0.0118 | 0 | 3.1 |
| 79..81 | 0.003 | 722.9 | 261.1 | 94.5401 | 0.0014 | 0 | 1 | 0 |
| 81..82 | 0.008 | 722.9 | 261.1 | 0.9335 | 0.0027 | 0.0029 | 1.9 | 0.8 |
| 82..83 | 0.074 | 722.9 | 261.1 | 0.2396 | 0.0133 | 0.0556 | 9.6 | 14.5 |
| 83..8 | 0.004 | 722.9 | 261.1 | 91.7679 | 0.0018 | 0 | 1.3 | 0 |
| 83..19 | 0.081 | 722.9 | 261.1 | 0.7372 | 0.0246 | 0.0334 | 17.8 | 8.7 |
| 82..18 | 0.079 | 722.9 | 261.1 | 0.4321 | 0.0196 | 0.0452 | 14.1 | 11.8 |
| 81..17 | 0.025 | 722.9 | 261.1 | 0.3597 | 0.0057 | 0.0158 | 4.1 | 4.1 |
| 51..14 | 0.022 | 722.9 | 261.1 | 0.0598 | 0.0014 | 0.0235 | 1 | 6.1 |
| 50..44 | 0.025 | 722.9 | 261.1 | 1.0876 | 0.0085 | 0.0078 | 6.1 | 2 |
| 49..84 | 0.008 | 722.9 | 261.1 | 0.2372 | 0.0014 | 0.0059 | 1 | 1.5 |
| 84..11 | 0.008 | 722.9 | 261.1 | 0.3872 | 0.0018 | 0.0047 | 1.3 | 1.2 |
| 84..15 | 0.008 | 722.9 | 261.1 | 0.2407 | 0.0014 | 0.0058 | 1 | 1.5 |
| 48..33 | 0.087 | 722.9 | 261.1 | 0.4307 | 0.0214 | 0.0496 | 15.4 | 12.9 |
| 47..85 | 0.003 | 722.9 | 261.1 | 80.2116 | 0.0014 | 0 | 1 | 0 |
| 85..31 | 0 | 722.9 | 261.1 | 1.4274 | 0 | 0 | 0 | 0 |
| 85..32 | 0.009 | 722.9 | 261.1 | 148.5636 | 0.0042 | 0 | 3.1 | 0 |
| Tree length for dN=0.7950 | | |  |  |  |  |  |  |
| Tree length for dS=1.8633 | | |  |  |  |  |  |  |

| **Table S8. Population genetic estimates polymorphism and divergence in *hcpG* and *hcpC*** | | | | | | | | |
| --- | --- | --- | --- | --- | --- | --- | --- | --- |
|  |  |  |  |  |  |  |  |  |
| **Gene** | **π/site** | **θ^W^/site (from S)** | **k** | **θ^W^/sequence (from S)** | **π_S_** | **π_A_** | **π_A_/π_S_ = ω_π_** |  |
| *hcpC* | 0.04571 | 0.04999 | 37.42 | 42.92 | 0.16647 | 0.016 | 0.096 |  |
| *hcpG* | 0.160641 | 0.11649 | 10.705 | 12.93 | 0.18231 | 0.0848 | 0.465 |  |

| **Table S9. Population differentiation and selection in *hcpC* measure by F_ST_^‡^ and α^‡‡,^ respectively.** | | | | | | | | | | |
| --- | --- | --- | --- | --- | --- | --- | --- | --- | --- | --- |
|  | **Shimaa** | | **East-Asia** | **Spain** | **Urban-Peru** | **The Gambia** |  |  |  |  |
| **Shimaa** | **-** | | **0.3971^†^** | **0.3996** | **0.53354** | **0.42138** |  |  |  |  |
| **East-Asia** | NC | | **-** | **0.42051** | **0.44757** | **0.54828** |  |  |  |  |
| **Spain** | 1 | | -0.035 | **-** | **0.0456** | **0.18769** |  |  |  |  |
| **Urban-Peru** | 0.568 | | NC | NC | **-** | **0.10118^¶^** |  |  |  |  |
| **The Gambia** | -1.667 | | -1.251 | NC | NC | **-** |  |  |  |  |
| **The upper triangle shows F_ST_ values:** | | | | | | |  |  |  |  |
| ‡, Calculated as described by Hudson, Boos Kaplan (1992) | | | | | | |  |  |  |  |
| †, Bold letters indicate significant values from the permutation tests (0.001<p<0.01) | | | | | | | | |  |  |
| ¶, 0.01<p<0.05 | |  | |  |  |  |  |  |  |  |
|  |  |  | |  |  |  |  |  |  |  |
| **The lower triangle shows α-values calculated as described by Fay et al (2001)** | | | | | | | | |  |  |
| ‡‡, α, estimates the proportion of codons under positive selection | | | | | | | |  |  |  |
| NC: Not calculated. In such cases a 2 X 2 contingency tables for the MacDonald-Kreitman test could not be constructed due to lack of nonsynonymous fixed differences between populations. | | | | | | | | | |  |

| **Table S10: dN/dS for each *hcpC* lineage computed by the M1bra model.** | | | | | | | | |
| --- | --- | --- | --- | --- | --- | --- | --- | --- |
| **Branch** | **t** | **N** | **S** | **dN/dS** | **dN** | **dS** | **N*dN** | **S*dS** |
| 82..1 | 0.014 | 611.3 | 231.7 | 0.3757 | 0.0033 | 0.0088 | 2 | 2 |
| 82..83 | 0.004 | 611.3 | 231.7 | 0.0001 | 0 | 0.0043 | 0 | 1 |
| 83..3 | 0.018 | 611.3 | 231.7 | 0.0935 | 0.0016 | 0.0176 | 1 | 4.1 |
| 83..84 | 0 | 611.3 | 231.7 | 0.0001 | 0 | 0 | 0 | 0 |
| 84..85 | 0.004 | 611.3 | 231.7 | 0.0001 | 0 | 0.0043 | 0 | 1 |
| 85..86 | 0.011 | 611.3 | 231.7 | 0.1849 | 0.0016 | 0.0089 | 1 | 2.1 |
| 86..87 | 0.018 | 611.3 | 231.7 | 0.2484 | 0.0033 | 0.0132 | 2 | 3.1 |
| 87..8 | 0.007 | 611.3 | 231.7 | 0.0001 | 0 | 0.0088 | 0 | 2 |
| 87..88 | 0 | 611.3 | 231.7 | 0.0001 | 0 | 0 | 0 | 0 |
| 88..89 | 0.011 | 611.3 | 231.7 | 0.7434 | 0.0033 | 0.0044 | 2 | 1 |
| 89..2 | 0.059 | 611.3 | 231.7 | 0.4095 | 0.0141 | 0.0343 | 8.6 | 8 |
| 89..4 | 0.004 | 611.3 | 231.7 | 0.0001 | 0 | 0.0043 | 0 | 1 |
| 88..90 | 0.011 | 611.3 | 231.7 | 0.0001 | 0 | 0.0132 | 0 | 3.1 |
| 90..14 | 0.004 | 611.3 | 231.7 | 433.3434 | 0.0016 | 0 | 1 | 0 |
| 90..91 | 0.007 | 611.3 | 231.7 | 0.0001 | 0 | 0.0087 | 0 | 2 |
| 91..6 | 0 | 611.3 | 231.7 | 0.362 | 0 | 0 | 0 | 0 |
| 91..12 | 0.004 | 611.3 | 231.7 | 0.0001 | 0 | 0.0043 | 0 | 1 |
| 86..92 | 0.022 | 611.3 | 231.7 | 0.0738 | 0.0016 | 0.0223 | 1 | 5.2 |
| 92..20 | 0.015 | 611.3 | 231.7 | 0.1212 | 0.0016 | 0.0135 | 1 | 3.1 |
| 92..21 | 0.033 | 611.3 | 231.7 | 0.466 | 0.0083 | 0.0177 | 5 | 4.1 |
| 86..93 | 0.023 | 611.3 | 231.7 | 0.0686 | 0.0016 | 0.024 | 1 | 5.6 |
| 93..94 | 0.019 | 611.3 | 231.7 | 0.0001 | 0 | 0.0225 | 0 | 5.2 |
| 94..95 | 0.005 | 611.3 | 231.7 | 0.0001 | 0 | 0.0058 | 0 | 1.4 |
| 95..96 | 0.004 | 611.3 | 231.7 | 0.0001 | 0 | 0.0043 | 0 | 1 |
| 96..97 | 0.004 | 611.3 | 231.7 | 0.0001 | 0 | 0.005 | 0 | 1.2 |
| 97..98 | 0.021 | 611.3 | 231.7 | 0.0001 | 0 | 0.0258 | 0 | 6 |
| 98..24 | 0.004 | 611.3 | 231.7 | 437.1775 | 0.0017 | 0 | 1 | 0 |
| 98..32 | 0.007 | 611.3 | 231.7 | 0.3794 | 0.0016 | 0.0043 | 1 | 1 |
| 97..99 | 0.004 | 611.3 | 231.7 | 433.0323 | 0.0017 | 0 | 1 | 0 |
| 99..100 | 0.011 | 611.3 | 231.7 | 0.1762 | 0.0017 | 0.0094 | 1 | 2.2 |
| 100..30 | 0.011 | 611.3 | 231.7 | 0.1864 | 0.0016 | 0.0088 | 1 | 2 |
| 100..80 | 0.007 | 611.3 | 231.7 | 0.0001 | 0 | 0.0088 | 0 | 2 |
| 99..101 | 0.01 | 611.3 | 231.7 | 0.0001 | 0 | 0.0125 | 0 | 2.9 |
| 101..71 | 0.022 | 611.3 | 231.7 | 0.7562 | 0.0066 | 0.0088 | 4.1 | 2 |
| 101..73 | 0 | 611.3 | 231.7 | 0.0001 | 0 | 0 | 0 | 0 |
| 96..102 | 0.007 | 611.3 | 231.7 | 0.0001 | 0 | 0.0088 | 0 | 2 |
| 102..75 | 0.022 | 611.3 | 231.7 | 0.3825 | 0.005 | 0.013 | 3 | 3 |
| 102..103 | 0.004 | 611.3 | 231.7 | 0.0001 | 0 | 0.0043 | 0 | 1 |
| 103..104 | 0.014 | 611.3 | 231.7 | 0.1251 | 0.0017 | 0.0132 | 1 | 3.1 |
| 104..31 | 0.004 | 611.3 | 231.7 | 438.4676 | 0.0017 | 0 | 1 | 0 |
| 104..59 | 0.007 | 611.3 | 231.7 | 551.5752 | 0.0033 | 0 | 2 | 0 |
| 103..105 | 0.004 | 611.3 | 231.7 | 0.0001 | 0 | 0.0048 | 0 | 1.1 |
| 105..74 | 0.025 | 611.3 | 231.7 | 0.0001 | 0 | 0.0307 | 0 | 7.1 |
| 105..106 | 0.011 | 611.3 | 231.7 | 0.1893 | 0.0017 | 0.0087 | 1 | 2 |
| **Branch** | **t** | **N** | **S** | **dN/dS** | **dN** | **dS** | **N*dN** | **S*dS** |
| 106..44 | 0.011 | 611.3 | 231.7 | 0.0001 | 0 | 0.0135 | 0 | 3.1 |
| 106..45 | 0.029 | 611.3 | 231.7 | 0.2254 | 0.005 | 0.022 | 3 | 5.1 |
| 95..107 | 0.004 | 611.3 | 231.7 | 0.0001 | 0 | 0.0044 | 0 | 1 |
| 107..43 | 0.018 | 611.3 | 231.7 | 0.0939 | 0.0017 | 0.0176 | 1 | 4.1 |
| 107..108 | 0.011 | 611.3 | 231.7 | 0.188 | 0.0017 | 0.0088 | 1 | 2 |
| 108..29 | 0.014 | 611.3 | 231.7 | 0.3783 | 0.0033 | 0.0087 | 2 | 2 |
| 108..81 | 0.029 | 611.3 | 231.7 | 0.1249 | 0.0033 | 0.0265 | 2 | 6.1 |
| 94..109 | 0.005 | 611.3 | 231.7 | 0.0001 | 0 | 0.0056 | 0 | 1.3 |
| 109..72 | 0.049 | 611.3 | 231.7 | 0.3026 | 0.01 | 0.033 | 6.1 | 7.6 |
| 109..110 | 0.018 | 611.3 | 231.7 | 0.0916 | 0.0017 | 0.0181 | 1 | 4.2 |
| 110..28 | 0.044 | 611.3 | 231.7 | 0.0337 | 0.0016 | 0.0486 | 1 | 11.3 |
| 110..61 | 0.023 | 611.3 | 231.7 | 0.3365 | 0.005 | 0.0148 | 3 | 3.4 |
| 93..111 | 0.043 | 611.3 | 231.7 | 0.2802 | 0.0083 | 0.0297 | 5.1 | 6.9 |
| 111..112 | 0.023 | 611.3 | 231.7 | 0.3503 | 0.005 | 0.0142 | 3 | 3.3 |
| 112..113 | 0.021 | 611.3 | 231.7 | 0.3406 | 0.0045 | 0.0133 | 2.8 | 3.1 |
| 113..114 | 0.027 | 611.3 | 231.7 | 0.0549 | 0.0015 | 0.0282 | 0.9 | 6.5 |
| 114..115 | 0.018 | 611.3 | 231.7 | 0.0001 | 0 | 0.0218 | 0 | 5 |
| 115..116 | 0.035 | 611.3 | 231.7 | 1.8847 | 0.0133 | 0.007 | 8.1 | 1.6 |
| 116..25 | 0.04 | 611.3 | 231.7 | 0.0376 | 0.0016 | 0.0437 | 1 | 10.1 |
| 116..41 | 0.072 | 611.3 | 231.7 | 0.0939 | 0.0066 | 0.0703 | 4 | 16.3 |
| 115..117 | 0.019 | 611.3 | 231.7 | 0.0872 | 0.0016 | 0.0189 | 1 | 4.4 |
| 117..60 | 0.022 | 611.3 | 231.7 | 0.3751 | 0.0049 | 0.0132 | 3 | 3.1 |
| 117..118 | 0.008 | 611.3 | 231.7 | 0.0001 | 0 | 0.0099 | 0 | 2.3 |
| 118..26 | 0.077 | 611.3 | 231.7 | 0.3015 | 0.0157 | 0.052 | 9.6 | 12.1 |
| 118..119 | 0.007 | 611.3 | 231.7 | 459.265 | 0.0033 | 0 | 2 | 0 |
| 119..35 | 0.058 | 611.3 | 231.7 | 0.0531 | 0.0033 | 0.0622 | 2 | 14.4 |
| 119..58 | 0.031 | 611.3 | 231.7 | 0.0501 | 0.0016 | 0.0329 | 1 | 7.6 |
| 119..120 | 0.035 | 611.3 | 231.7 | 0.2697 | 0.0066 | 0.0246 | 4 | 5.7 |
| 120..27 | 0.04 | 611.3 | 231.7 | 0.2145 | 0.0066 | 0.0308 | 4 | 7.1 |
| 120..121 | 0.014 | 611.3 | 231.7 | 0.127 | 0.0016 | 0.0129 | 1 | 3 |
| 121..50 | 0.046 | 611.3 | 231.7 | 0.0697 | 0.0033 | 0.0471 | 2 | 10.9 |
| 121..122 | 0.021 | 611.3 | 231.7 | 0.0001 | 0 | 0.0253 | 0 | 5.9 |
| 122..49 | 0.036 | 611.3 | 231.7 | 0.4351 | 0.0089 | 0.0206 | 5.5 | 4.8 |
| 122..123 | 0.015 | 611.3 | 231.7 | 0.0001 | 0 | 0.0176 | 0 | 4.1 |
| 123..55 | 0 | 611.3 | 231.7 | 0.0001 | 0 | 0 | 0 | 0 |
| 123..69 | 0.004 | 611.3 | 231.7 | 0.0001 | 0 | 0.0043 | 0 | 1 |
| 114..124 | 0.012 | 611.3 | 231.7 | 0.0001 | 0 | 0.0149 | 0 | 3.5 |
| 124..37 | 0.042 | 611.3 | 231.7 | 0.276 | 0.0082 | 0.0298 | 5 | 6.9 |
| 124..47 | 0.047 | 611.3 | 231.7 | 0.1135 | 0.0049 | 0.0435 | 3 | 10.1 |
| 113..125 | 0.006 | 611.3 | 231.7 | 0.0001 | 0 | 0.0074 | 0 | 1.7 |
| 125..79 | 0.079 | 611.3 | 231.7 | 0.1143 | 0.0084 | 0.0732 | 5.1 | 16.9 |
| 125..126 | 0.013 | 611.3 | 231.7 | 0.0001 | 0 | 0.0158 | 0 | 3.7 |
| 126..62 | 0.038 | 611.3 | 231.7 | 0.0849 | 0.0032 | 0.0375 | 1.9 | 8.7 |
| 126..78 | 0.036 | 611.3 | 231.7 | 0.0451 | 0.0017 | 0.0386 | 1.1 | 8.9 |
| 112..127 | 0.023 | 611.3 | 231.7 | 0.0701 | 0.0016 | 0.0234 | 1 | 5.4 |
| **Branch** | **t** | **N** | **S** | **dN/dS** | **dN** | **dS** | **N*dN** | **S*dS** |
| 127..65 | 0.059 | 611.3 | 231.7 | 0.0525 | 0.0033 | 0.0632 | 2 | 14.6 |
| 127..128 | 0.006 | 611.3 | 231.7 | 0.0001 | 0 | 0.0068 | 0 | 1.6 |
| 128..129 | 0.024 | 611.3 | 231.7 | 0.0001 | 0 | 0.0293 | 0 | 6.8 |
| 129..66 | 0.081 | 611.3 | 231.7 | 0.3123 | 0.0168 | 0.0537 | 10.3 | 12.4 |
| 129..130 | 0.006 | 611.3 | 231.7 | 0.0001 | 0 | 0.0067 | 0 | 1.6 |
| 130..64 | 0.02 | 611.3 | 231.7 | 0.0832 | 0.0017 | 0.0201 | 1 | 4.6 |
| 130..131 | 0.036 | 611.3 | 231.7 | 0.041 | 0.0016 | 0.0399 | 1 | 9.2 |
| 131..33 | 0.057 | 611.3 | 231.7 | 0.0882 | 0.0049 | 0.0561 | 3 | 13 |
| 131..39 | 0.037 | 611.3 | 231.7 | 0.243 | 0.0066 | 0.0273 | 4.1 | 6.3 |
| 128..132 | 0.012 | 611.3 | 231.7 | 0.5512 | 0.0033 | 0.006 | 2 | 1.4 |
| 132..51 | 0.078 | 611.3 | 231.7 | 0.0866 | 0.0066 | 0.0766 | 4.1 | 17.7 |
| 132..67 | 0.052 | 611.3 | 231.7 | 0.0987 | 0.005 | 0.0503 | 3 | 11.6 |
| 112..133 | 0.012 | 611.3 | 231.7 | 0.1613 | 0.0017 | 0.0103 | 1 | 2.4 |
| 133..42 | 0.048 | 611.3 | 231.7 | 0.1205 | 0.0053 | 0.0441 | 3.3 | 10.2 |
| 133..134 | 0.034 | 611.3 | 231.7 | 0.045 | 0.0016 | 0.0366 | 1 | 8.5 |
| 134..48 | 0.045 | 611.3 | 231.7 | 0.119 | 0.0049 | 0.0416 | 3 | 9.6 |
| 134..56 | 0.041 | 611.3 | 231.7 | 0.0806 | 0.0033 | 0.0409 | 2 | 9.5 |
| 112..135 | 0.013 | 611.3 | 231.7 | 0.0001 | 0 | 0.0154 | 0 | 3.6 |
| 135..136 | 0.025 | 611.3 | 231.7 | 0.0001 | 0 | 0.0306 | 0 | 7.1 |
| 136..68 | 0.048 | 611.3 | 231.7 | 0.066 | 0.0033 | 0.0498 | 2 | 11.5 |
| 136..137 | 0.025 | 611.3 | 231.7 | 0.1509 | 0.0033 | 0.022 | 2 | 5.1 |
| 137..46 | 0.048 | 611.3 | 231.7 | 0.1096 | 0.005 | 0.0454 | 3 | 10.5 |
| 137..53 | 0.011 | 611.3 | 231.7 | 0.1876 | 0.0016 | 0.0087 | 1 | 2 |
| 135..138 | 0.019 | 611.3 | 231.7 | 0.0001 | 0 | 0.0229 | 0 | 5.3 |
| 138..57 | 0.044 | 611.3 | 231.7 | 0.1235 | 0.005 | 0.0401 | 3 | 9.3 |
| 138..76 | 0.026 | 611.3 | 231.7 | 0.061 | 0.0017 | 0.0271 | 1 | 6.3 |
| 111..139 | 0.017 | 611.3 | 231.7 | 0.2766 | 0.0033 | 0.0119 | 2 | 2.8 |
| 139..140 | 0.005 | 611.3 | 231.7 | 0.0001 | 0 | 0.0061 | 0 | 1.4 |
| 140..141 | 0.018 | 611.3 | 231.7 | 0.0978 | 0.0017 | 0.0169 | 1 | 3.9 |
| 141..34 | 0.049 | 611.3 | 231.7 | 0.2166 | 0.0083 | 0.0381 | 5 | 8.8 |
| 141..142 | 0.032 | 611.3 | 231.7 | 0.047 | 0.0016 | 0.0348 | 1 | 8.1 |
| 142..54 | 0 | 611.3 | 231.7 | 0.0923 | 0 | 0 | 0 | 0 |
| 142..70 | 0.007 | 611.3 | 231.7 | 0.3759 | 0.0016 | 0.0043 | 1 | 1 |
| 140..143 | 0.01 | 611.3 | 231.7 | 0.204 | 0.0016 | 0.0081 | 1 | 1.9 |
| 143..36 | 0.038 | 611.3 | 231.7 | 0.0001 | 0 | 0.0464 | 0 | 10.7 |
| 143..77 | 0.047 | 611.3 | 231.7 | 0.2339 | 0.0083 | 0.0353 | 5.1 | 8.2 |
| 139..144 | 0.013 | 611.3 | 231.7 | 0.1486 | 0.0016 | 0.0111 | 1 | 2.6 |
| 144..63 | 0.046 | 611.3 | 231.7 | 0.0001 | 0 | 0.0561 | 0 | 13 |
| 144..145 | 0.006 | 611.3 | 231.7 | 0.6532 | 0.0016 | 0.0025 | 1 | 0.6 |
| 145..40 | 0.066 | 611.3 | 231.7 | 0.0001 | 0 | 0.0797 | 0 | 18.5 |
| 145..146 | 0.023 | 611.3 | 231.7 | 0.0705 | 0.0016 | 0.0232 | 1 | 5.4 |
| 146..38 | 0.05 | 611.3 | 231.7 | 0.0641 | 0.0033 | 0.0514 | 2 | 11.9 |
| 146..52 | 0.033 | 611.3 | 231.7 | 0.0455 | 0.0016 | 0.0362 | 1 | 8.4 |
| 85..147 | 0.011 | 611.3 | 231.7 | 0.1846 | 0.0016 | 0.0089 | 1 | 2.1 |
| 147..148 | 0.007 | 611.3 | 231.7 | 0.3767 | 0.0016 | 0.0044 | 1 | 1 |
| **Branch** | **t** | **N** | **S** | **dN/dS** | **dN** | **dS** | **N*dN** | **S*dS** |
| 148..10 | 0.007 | 611.3 | 231.7 | 547.9555 | 0.0033 | 0 | 2 | 0 |
| 148..17 | 0.004 | 611.3 | 231.7 | 0.0001 | 0 | 0.0044 | 0 | 1 |
| 148..18 | 0.018 | 611.3 | 231.7 | 0.565 | 0.0049 | 0.0088 | 3 | 2 |
| 148..149 | 0.006 | 611.3 | 231.7 | 0.4904 | 0.0016 | 0.0034 | 1 | 0.8 |
| 149..16 | 0.006 | 611.3 | 231.7 | 0.0001 | 0 | 0.0071 | 0 | 1.6 |
| 149..150 | 0.006 | 611.3 | 231.7 | 0.0001 | 0 | 0.0071 | 0 | 1.6 |
| 150..5 | 0 | 611.3 | 231.7 | 0.1252 | 0 | 0 | 0 | 0 |
| 150..11 | 0.004 | 611.3 | 231.7 | 0.0001 | 0 | 0.0043 | 0 | 1 |
| 147..151 | 0.01 | 611.3 | 231.7 | 0.8453 | 0.0033 | 0.0039 | 2 | 0.9 |
| 151..13 | 0.011 | 611.3 | 231.7 | 0.1779 | 0.0016 | 0.0092 | 1 | 2.1 |
| 151..15 | 0.041 | 611.3 | 231.7 | 0.0362 | 0.0016 | 0.0455 | 1 | 10.5 |
| 84..152 | 0.022 | 611.3 | 231.7 | 0.1867 | 0.0033 | 0.0177 | 2 | 4.1 |
| 152..9 | 0.025 | 611.3 | 231.7 | 0.2788 | 0.005 | 0.0178 | 3 | 4.1 |
| 152..153 | 0.007 | 611.3 | 231.7 | 0.3826 | 0.0016 | 0.0043 | 1 | 1 |
| 153..19 | 0.004 | 611.3 | 231.7 | 0.0001 | 0 | 0.0043 | 0 | 1 |
| 153..22 | 0.011 | 611.3 | 231.7 | 625.3174 | 0.005 | 0 | 3 | 0 |
| 82..154 | 0.007 | 611.3 | 231.7 | 0.3771 | 0.0016 | 0.0044 | 1 | 1 |
| 154..7 | 0.025 | 611.3 | 231.7 | 0.9369 | 0.0083 | 0.0088 | 5.1 | 2 |
| 154..23 | 0.011 | 611.3 | 231.7 | 0.1878 | 0.0016 | 0.0088 | 1 | 2 |
| tree length for dN: 0.3990 | | | | |  |  |  |  |
| tree length for dS: 3.0255 | | | |  |  |  |  |  |
